# Unsupervised Methods for Detection of Neural States: Case Study of Hippocampal-Amygdala Interactions

**DOI:** 10.1523/ENEURO.0484-20.2021

**Published:** 2021-11-02

**Authors:** Francesco Cocina, Andreas Vitalis, Amedeo Caflisch

**Affiliations:** Biochemistry department, University of Zurich, Zurich, Switzerland CH-8057

**Keywords:** amygdala, hippocampus, interactions, local field potential, neural states, unsupervised methods

## Abstract

The hippocampus and amygdala are functionally coupled brain regions that play a crucial role in processes involving memory and learning. Because interareal communication has been reported both during specific sleep stages and in awake, behaving animals, these brain regions can serve as an archetype to establish that measuring functional interactions is important for comprehending neural systems. To this end, we analyze here a public dataset of local field potentials (LFPs) recorded in rats simultaneously from the hippocampus and amygdala during different behaviors. Employing a specific, time-lagged embedding technique, named topological causality (TC), we infer directed interactions between the LFP band powers of the two regions across six frequency bands in a time-resolved manner. The combined power and interaction signals are processed with our own unsupervised tools developed originally for the analysis of molecular dynamics simulations to effectively visualize and identify putative, neural states that are visited by the animals repeatedly. Our proposed methodology minimizes impositions onto the data, such as isolating specific epochs, or averaging across externally annotated behavioral stages, and succeeds in separating internal states by external labels such as sleep or stimulus events. We show that this works better for two of the three rats we analyzed, and highlight the need to acknowledge individuality in analyses of this type. Importantly, we demonstrate that the quantification of functional interactions is a significant factor in discriminating these external labels, and we suggest our methodology as a general tool for large, multisite recordings.

## Significance Statement

We develop an analysis pipeline for neuroscience datasets. We test it on a published example of multielectrode recordings of rats in a range of behaviors: running on a track, sleeping, collecting rewards, etc. We adopt nonlinear analysis techniques that are able to quantify directed interactions between different signals, here oscillations of two brain regions in different frequency bands. Using the entire recordings and, importantly, distinguishing each animal, we provide a high-resolution overview of the functional interplay of the two regions. Putative neural states that the animals can be in are derived from a time-aware clustering of the large datasets. When discriminating experimental annotations like run speed, we provide evidence that our methodology outperforms common clustering techniques.

## Introduction

A variety of oscillation-related phenomena have been identified within the hippocampus and amygdala, and they are frequently associated with various learning and memory processes that strongly rely on these two brain structures (Paré et al., 2002; Buzsáki and Draguhn, 2004; Colgin, 2016). The coordination between the local field potentials (LFPs) of hippocampus and amygdala, in particular the basolateral complex [basolateral amygdala (BLA)], was highlighted and shown to involve different frequency ranges and interaction modalities (Bocchio et al., 2017; Pesaran et al., 2018). Various studies investigated a number of stimuli and behavioral aspects related to emotional processing, in particular connected to fear and safety, both in rodents (Seidenbecher et al., 2003; Popa et al., 2010; Likhtik et al., 2014; Stujenske et al., 2014) as well as in epileptic patients (Zheng et al., 2017, 2019; Kirkby et al., 2018). These analyses often focus on selected time periods, such as rapid eye movement (REM) sleep or behavioral stages in experiments relying on conditioned fear, and they investigate specific questions of memory-related processes, such as consolidation or retrieval, in specific frequency bands (Bocchio et al., 2017).

All of these results suggest, as a whole, persistent yet diverse ways in which hippocampus and BLA interact with each other, and this set of coupled regions can be extended to other parts of the brain, such as prefrontal or entorhinal cortex (Bauer et al., 2007; Stujenske et al., 2014). Clearly, these interactions might extend beyond the specific cases observed so far in the literature. Indeed, more recent studies shed light on additional functions of both hippocampus and amygdala (Sawangjit et al., 2018; Gründemann et al., 2019; Schapiro et al., 2019), thus broadening the scope for the functions of interareal communication patterns. To arrive at and/or test new hypotheses, it would thus be helpful to collect a global picture of this communication flow where no restrictions to specific time frames or features are imposed, as well as to explore new workflows to achieve this goal in an efficient manner. The potential for new discoveries can, in principle, be maximized if all the information in the time series is preserved. This stipulates that averaging operations, for example, across time windows, sessions, or animals, should be avoided as they may mask variance of interest.

**Figure 1.**
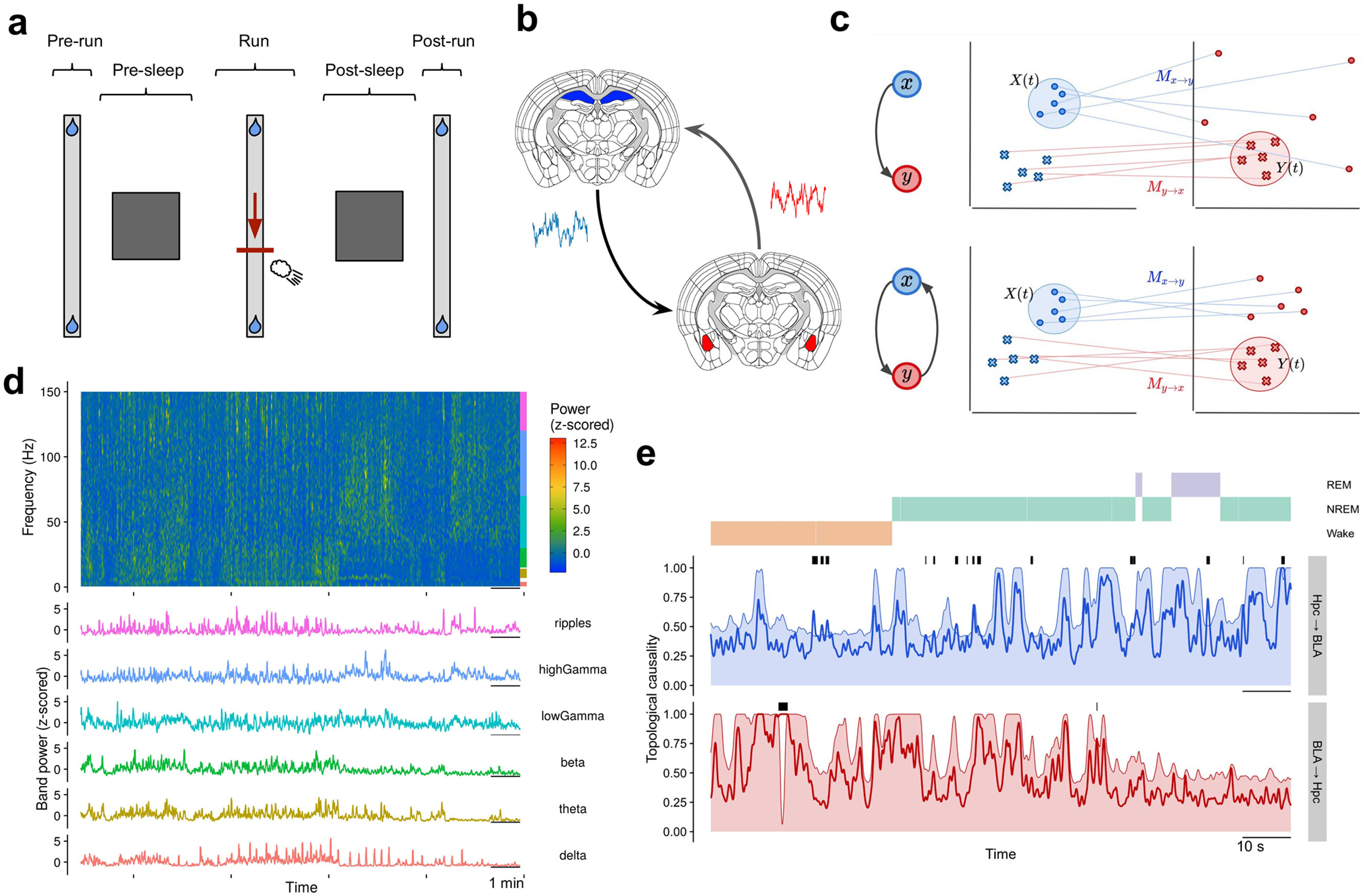
Experiment, hippocampus-BLA interactions, and TC. ***A***, Description of the experiment. In the pre-run epoch (left), the rat runs back and forth freely on the linear track, receiving water rewards at both ends (∼3 min each). Successively, the animal is located in a separate environment for sleeping (∼2–3 h). In the run epoch (middle), back on the linear track, the rat experiences an air puff in a fixed location but only when moving in one specific direction (∼30 min). This direction and the precise location of the air puff are set randomly. The session is terminated by post-sleep and post-run epochs (right), which have the same modalities of the pre-sleep and pre-run epochs, respectively. ***B***, LFPs are recorded in the dorsal hippocampus and in the BLA. Only LFP oscillations from the right BLA are used. ***C***, Illustration of the TC method. Two different scenarios of interaction are shown. In the upper panels, *x* driving *y* unilaterally implies that the mapping *M_y_*_→_*_x_* preserves the local neighborhood of *Y* (the embedded space of *y*) when projected onto *X* (the embedded space of *x*). Conversely, when a neighborhood in *X* is projected onto *Y*, the points are scattered over the whole dynamical space. This is because *x* evolves independently of the dynamics of *y*, which implies that *M_x_*_→_*_y_* is ill-defined (its Jacobian diverges everywhere). Instead, when the interactions are bidirectional (lower panel), *X* contains information about *Y*, so that *M_x_*_→_*_y_* is well-defined and preserves the local neighborhood of *X* when mapped to *Y*. ***D***, Spectogram of 15-min recordings in the hippocampus (top) along with the six different band powers extracted from it (bottom). Frequency ranges are indicated by colored bars on the side of the spectrogram (ripples band extends up to 250 Hz). Power densities in the spectrogram are z-scored within each frequency bin. The band powers (bottom panel) are used to calculate the TC values. ***E***, An example of a TC time series of ∼90 s across different sleep phases (color labels on top) in both directions of putative influence. The actual values (solid lines) exceed the chance level (shaded areas, 95% quantile from shuffling test, see Materials and Methods, Topological causality) only for short, sporadic intervals (black bars on top of the time series). In the final TC time series, non-significant values are zeroed explicitly. The full description of the TC method is provided in Materials and Methods, Topological causality. The alignment of the embeddings of the two time series of band powers is described in Materials and Methods, Alignment settings, and shown in Extended Data Figure 1-1.

In this work we propose a data-driven procedure to explore an extensive dataset of recordings of LFPs and unveil potential neural states in the context of interactions between regions. The unsupervised procedure is applied here in the well-studied hippocampus-amygdala system, but it can be applied equally well to other areas of the brain. We use a dataset of simultaneous recordings in the rat hippocampus and BLA (Girardeau et al., 2017a), publicly available on https://crcns.org/ (Girardeau et al., 2017b), which comprises multiple behavioral epochs. In these experiments, rats run on a linear track with water rewards placed at the track’s ends. A negative (aversive) stimulus in the form of an air puff is delivered in the run epoch and only when the animal travels along one of the two directions (Fig. 1*A*). During the non-REM (NREM) sleep phases following each run epoch, reactivations of joint neural activity of the hippocampus and the BLA were found to be modulated by hippocampal sharp-wave ripples (SWRs). Moreover, these reactivations were more pronounced for signals corresponding to the direction where the rat faced the aversive stimulus (Girardeau et al., 2017a). The aforementioned results corroborate the hypothesis that contextual, emotional memories derive from the integration of hippocampal neural activity, which encodes the spatial information, with activity in the amygdala, which assigns an emotional value to specific events (Phillips and LeDoux, 1992; Zelikowsky et al., 2014; Beyeler et al., 2016; Kim and Payne, 2020), and shed light on new mechanisms through which the two brain regions influence each other.

10.1523/ENEURO.0484-20.2021.f1-1 Extended Data Figure 1-1 Embedded time series and alignment settings. We illustrate here the alignment settings between TC, spectrograms, and external labels described in Materials and Methods, Alignment settings between TC, band powers, and external labels. For a given point in time, the “cursor” t, we show the spectrogram windows (in orange), and two time-lagged embeddings with dimension m = 3. The time lag between points in the embedding, τ, is dependent on the underlying time series but m is homogenized to the larger of the two values suggested by the simplex projection method (see Materials and Methods, Topological causality). For the TC calculation, the two embedded time series are aligned to each other to the right. We assign to the computed TC value the time t corresponding to the middle of the spectrogram window (thick dotted line). Note that this last operation is relevant only when comparing TC to other time-series characterizing events or behavioral stages, e.g., air puffs, SWRs, awake phase. The implications of these choices are analyzed in Materials and Methods, Alignment settings between TC, band powers, and external labels and Extended Data Figures 3-3, 3-4. Download Figure 1-1, EPS file.

We conducted a multistage exploratory analysis of directed interactions between hippocampus and BLA and of neural states associated with both activity and functional connectivity patterns. The analysis focuses on the mutual influence between LFP band powers in six different frequency ranges across the two brain regions, and it can be partitioned into three major steps. First, a standard comparison of average interaction values is conducted between relevant behavioral and physiological epochs. Among the different interaction patterns, θ oscillations and SWRs in particular carry strong signatures that distinguish different epochs. Second, the information provided by the interactions and LFP power is visualized at maximal time resolution on the global dataset, i.e., without restricting the search to specific epochs or features. We highlight four distinct patterns in these features that are qualitatively identifiable across three different rats. Third, an identification of distinct clusters, or states, in the hippocampus-BLA system is performed in an unsupervised way. More precisely, each state corresponds to a specific pattern that combines both the internal activity within each anatomical area, represented by the LFPs, and the ongoing reciprocal interactions between the two regions. We show that the contribution of interactions plays a significant role for the quality with which such states explain the, for example, behavioral labels that the experiments are annotated with.

The full description of the dataset and techniques is found in Materials and Methods, and the outcomes of the three-stage procedure are presented in Results. For ease of reading, the Results section also includes conceptual explanations of our methodology, both in pictorial and in text form. The analysis yields both expected and unexpected insights, and in the Discussion section we provide an appraisal of these insights both in terms of our chosen methodology and in terms of the biology of the hippocampus-amygdala system.

## Materials and Methods

### Experimental design and behavior

A full description of the experiment is given in the original article (Girardeau et al., 2017a). Here, we merely recapitulate the salient aspects that concern our analysis. Four male Long-Evans rats were trained to run back and forth on a linear track to obtain water rewards placed at both ends. Three silicon probes were implanted above the amygdala, left and right, and in the dorsal CA1 hippocampal region. The probes in the hippocampus had four shanks with 32 recording channels in total (NeuroNexus H32, A-style, Buzsaki32 design). The amygdala electrodes featured eight shanks with 64 channels in total (NeuroNexus H64, A-style, Buzsaki64 design) and were lowered by 140 μm every day at the end of each session. Only LFPs from the right BLA were used in the analysis; the number of selected channels recording in this region varied from a minimum of 24 to a maximum of 56. Recording sessions that exhibited low numbers of channels in the BLA, indicated signal degradation, or featured too few aversive stimuli (air puff events) were discarded. Eventually, we kept seven sessions for Rat1 and Rat2, and six for Rat3. The rats analyzed here coincide with the three animals, out of the original four, used in the reactivation analysis of Girardeau and colleagues. Specifically, Rat1, Rat2, and Rat3 here correspond, respectively, to Rat1, Rat3, and Rat4 of the published work (Girardeau et al., 2017a) as well as to Rat08, Rat10, and Rat11 in the online dataset (Girardeau et al., 2017b).

The position of the rat was tracked by the two LEDs attached to the head of the animal. Speed was computed as the average value between the two velocities but only when both LEDs were active. Safe trajectory periods were identified as those intervals between consecutive reward events, on opposite ends of the track, when no air puff was recorded. Conversely, we identified as “aversive” trajectories those intervals between a reward and an air puff event, or between multiple air puffs. The identification of different sleep phases (NREM/REM) was done with the help of a k-means clustering of the ratios of the δ to θ powers in the hippocampus (higher ratios point to NREM and vice versa), which we extracted from the spectrograms, see LFP preprocessing. The identification of NREM/REM was performed independently for each of the channels, and the final annotation was chosen as the consensus across channels. These data were restricted to periods of immobility, i.e., a sustained speed of below 3 cm/s for at least 30 s, with only brief (<0.5 s) stretches of exceeding this threshold being tolerated (Maingret et al., 2016; *QuietPeriods.m* and *Brainstates.m* in MATLAB toolbox FMAtoolbox). Results from spike sorting, units of classification (pyramidal/interneuron), and the SWR timestamps are provided in the original dataset. The latter were identified by the authors by filtering the hippocampal LFP between 100 and 200 Hz, then squaring and normalizing it (*z*-score; Girardeau et al., 2017a). SWR events were subsequently identified as time windows starting at 1 SD, peaking at >4 SD, and remaining at >1 SD for >20 and <130 ms around the peak.

### LFP preprocessing

Power bands were computed using multitaper spectograms (*MTSpectrogram.m* in FMAtoolbox) between 0 and 250 Hz with a window of 2 s, a step size of 50 ms, and a time-frequency bandwidth of three and five tapers (Purpura and Bokil, 2008; Babadi and Brown, 2014). The spectral density powers are estimated in the following frequency domains: δ (0.5–4 Hz), θ (7–14 Hz in hippocampus, 4–12 Hz in BLA), β (15–30 Hz in hippocampus, 12–30 Hz in BLA), low-γ (30–70 Hz), high-γ (70–120 Hz), and, eventually, “ripples” and “fast” (both 120–250 Hz) for hippocampus and BLA, respectively (Buzsáki and Draguhn, 2004; Girardeau and Zugaro, 2011; Colgin, 2016; Bocchio et al., 2017). Band powers were computed for all the electrodes recording in the selected anatomical areas, i.e., right BLA and hippocampus. The 85% quantile value of their distribution was computed and used throughout the analysis. Note that the mapping of band powers to absolute values of time is irrelevant as long as all power time series are treated the same and as long as no other time-resolved quantities are present. The latter becomes relevant below, see Alignment settings.

### Topological causality (TC)

TC relies on Takens’ theorem and the theories of time-lagged embedding that it forms the foundation of (Takens, 1981; Kantz and Schreiber, 2004; Bradley and Kantz, 2015; Harnack et al., 2017). A time-lagged embedding of dimensionality *m* refers to the procedure where a time series is converted to a sequence of vectors in an *m*-dimensional space, and each dimension is defined by a specific time lag (see below for the mathematical formulation). In simple terms, Takens’ theorem states that, given a partial observation of a dynamical system, e.g., of a time series of only one out of three effective variables in the system, a suitable embedding procedure will allow the (re)construction of an attractor preserving any true underlying attractor in a topological sense. This will hold if the dynamics are deterministic and couple the evolution of all underlying variables. TC exploits the theorem and its underlying theory, which is where the attribute “topological” derives from. Specifically, TC allows the quantification of the directed influence of two time series, which are assumed to be coupled with no requirement for linearity, on each other by essentially imposing Takens’ theorem and measuring the consistency of the data with it. The numerical method to estimate it closely follows the one suggested in the original study (Harnack et al., 2017). The actual implementation starts by transforming the data points in the time series under investigation into their empirical cumulative distribution values (this is referred to as a quantile transformation in Harnack et al., 2017). This is done to achieve invariance of the TCs with respect to the actual distributions of the underlying sequences. In our case, the time series, i.e., band powers, were transformed separately within each behavioral epoch.

The actual TC values are derived as follows. Given a scalar variable *x*, the *m*-dimensional reconstruction-space vectors are constructed as X(t)={x(t),x(t−τ),...,x(t−(m−1)τ)}. We chose the time lag *τ* as the 1/*e* characteristic decay value of the average mutual information (Fraser and Swinney, 1986; Bradley and Kantz, 2015). Next, Sugihara’s simplex projection method is applied to identify a suitable embedding dimension in a range of values from 2 to 10 (*simplex* in R package rEDM; Sugihara and May, 1990; Sugihara et al., 2012) where 10 turned out to be a sufficient upper limit in our case. *τ* and *m* are obtained independently for each of the two time series *x* and *y* under investigation. In the actual embedding for the evaluation of TCs, each time series maintains its own *τ* while the actual *m* used is the larger among the two (Cao et al., 1998; Arnhold et al., 1999; Hegger et al., 2000).

Given the embedded vector *X*(*t*), the *k *=* *2*m* nearest neighbors are found. Here, we applied also the Theiler correction that excludes the points belonging to a temporal neighborhood of radius *τ*(*m* – 1) from this search (Arnhold et al., 1999). The time indices of these *k* neighbors are extracted and used to identify the respective projection of *X* in the other embedded space *Y*. Let us denote as $K_{x}$ and $K_{y}$ the *k *×* m* matrices representing, respectively, the neighborhood of *X* and its projection in the embedded space of *Y*. We wish to compute the local Jacobian matrix $J_{y\to x}^{t}$ of the mapping from *y* to *x*. This is done by performing a principal component analysis (PCA) on the joint matrix $\left[K_{X},K_{Y}\right]$, followed by calculating $J_{y\to x}^{t}=P_{X}P_{Y}^{-1}$ where $P_{X}$( $P_{Y}$) are the projections of *X* (*Y*) onto the first *m* principal components. The singular values $\sigma_{k,y\to x}^{t}$ of the Jacobian matrix yield the TC components as follows:

(1) $$TC_{x\to y}^{t}=\frac{1}{1+\log\;(\prod_{k}\max(1,\sigma_{k,y\to x}^{t}))}.$$

In order to assess the statistical significance of the obtained value, we eliminate the notion of geometric neighborhood from the calculation. To do so, we select the *k* projections of *X* on *Y* randomly and recompute the TC value. By repeating this *N *=* *100 times, we are able to generate a chance level distribution in the TC domain [0, 1]. Because of the modest number of trials, which we were restricted to for computational reasons, a bounded density estimation with a β kernel and a bandwidth equal to *σN*^2/5^ (R package bde; Chen, 1999) was adopted for increasing robustness. The true TC value was deemed significant if it was larger than the 95% quantile of the chance level distribution, otherwise it was set to zero. The false discovery rate is controlled with the Benjamini–Yekutieli correction (Benjamini and Yekutieli, 2001), which was applied at every time step based on pooled data across the 72 TC indices (Sheikhattar et al., 2018).

The reconstruction of the manifold and the related computation of TC values presented below were performed within non-overlapping windows of 2-min length. The amount of independent data points depends on the rate of memory decay of the signals, and in our case the chosen interval of two minutes offered ∼100 observations (see below). The details of the localization are also relevant for controlling the impact of non-stationarity issues along the trajectory, which can arise because of transient events, such as extraneous stimuli or experimenter intervention (Pesaran et al., 2018). This is particularly relevant in the run epoch where heterogeneous behaviors are present, but we refrained from attempting to investigate this quantitatively.

### Alignment settings

Calculating the TCs and examining their values within specific time periods necessitated, to some degree, arbitrary choices regarding the temporal alignment between the different time series, namely, the band powers, TCs, and labels, e.g., NREM or SWRs. If we take the labels as reference for absolute time, this leaves two alignment choices to be made. Band powers were computed as multitaper spectrograms with windows of 2-s length, which were always center-aligned to the labels. The TC values, on their own, were computed in a lagged coordinate space, which corresponds to choosing a predictive embedding, i.e., for a given time point, the lagged data were taken exclusively from earlier times (for a pictorial description, see Extended Data Fig. 1-1). Clearly, the construction of spectrograms performs an averaging operation that will lead to some blurring in the localization of interactions. This effect should be kept in mind throughout. To investigate whether the analysis is robust with respect to the choice of alignment for these computed TC values, different alignments were analyzed systematically. TC embeddings were shifted, forward or backward, with respect to the time bins, and, thus, to the labels and power band windows. For each time bin, the 75% quantile over the 36 TCs was computed and, eventually, these values were averaged over the single sessions. As a global measure, the average of the unsigned differences between different time periods was computed (Extended Data Figs. 3-3, 3-4). Chance level distributions for each session were obtained by 1000 random block permutations of the TC values along the time axis (see below, Granger causality and cross-correlation functions), and the mean of the 95% quantiles across sessions was calculated.

**Figure 2.**
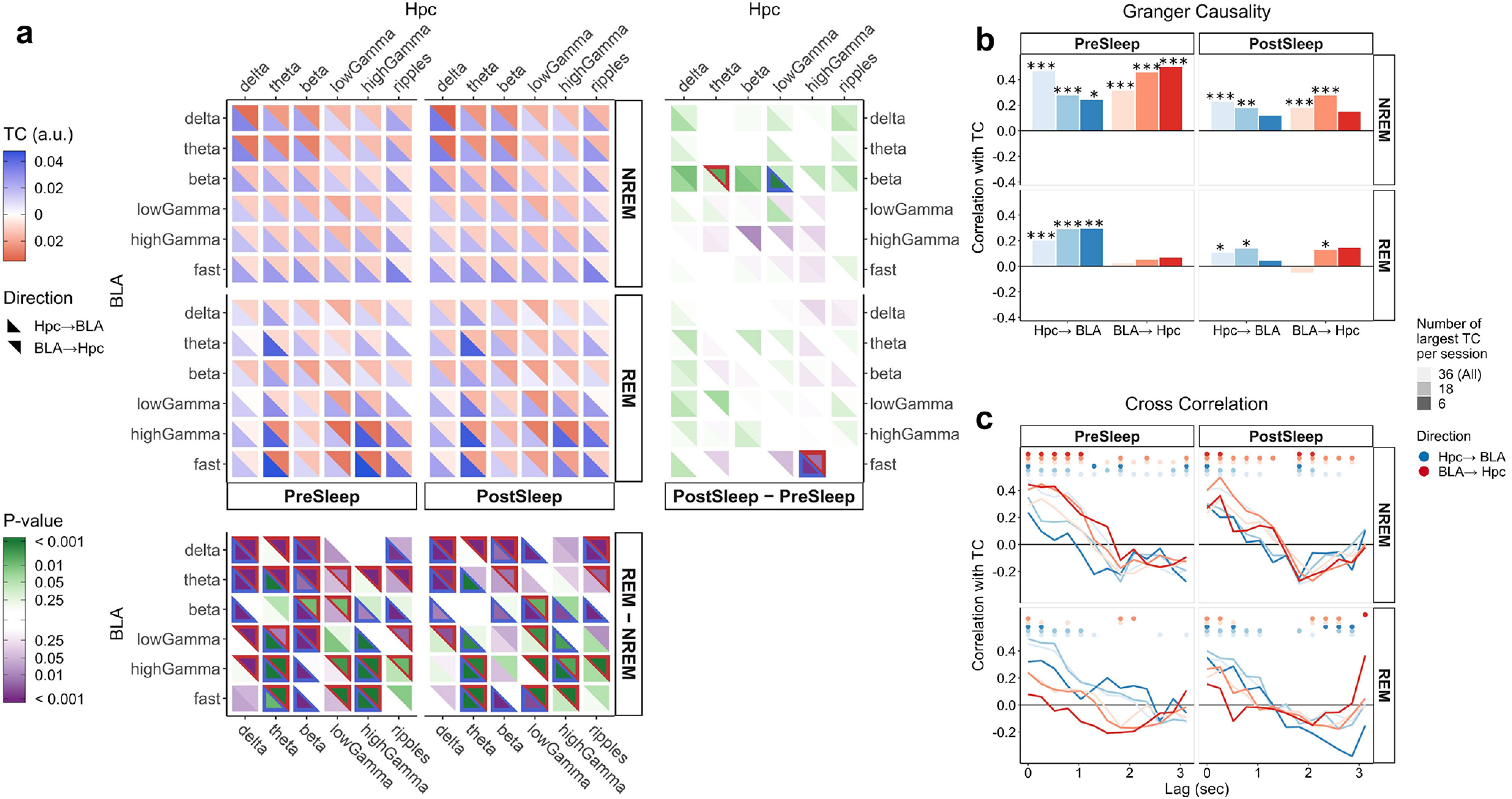
Reciprocal influence of hippocampus and BLA during sleep. ***A***, TC results in interaction tables. Central panels, Mean values across all the sessions (*n *=* *16, 3 rats) of the average TC values per sleep epoch and the two annotated sleep phases (NREM and REM). All sessions included in the calculation feature at least 1 min of total REM sleep in both pre-sleep and post-sleep phases. Every square shows two values for a given combination of frequency bands: the directions of influence are indicated by both color and placement of the two triangles (blue and lower-left for hippocampus → BLA, red and upper-right for the reverse). To aid visibility, the minimum value across the four panels and both directions was subtracted for plotting. Bottom and right panels, *p* values of the two-tailed Wilcoxon signed-rank test on the differences of the mean values shown in the central panels. The right panels show test results for differences between sleep epochs, and the bottom panels those for differences between sleep phases. Color hue is given by the sign of the test statistic (value of zero under the null hypothesis). Given a comparison of the type *A*–*B*, green colors indicate higher values in *A* relative to *B*. The triangle shapes are outlined when the corresponding *p* < 0.05. NREM was distinguished from REM sleep by the hippocampal δ/θ ratio (see Materials and Methods, Experimental design and behavior). The same analysis was performed for pre-run and post-run epochs when distinguishing between safe and aversive direction (Extended Data Fig. 2-1). To examine a possible impact of volume conduction on TC values, phase differences between the LFPs of the two areas are shown in Extended Data Figure 2-2. ***B***, Comparison of TC with GC. For each combination of sleep phases, epochs, and direction of influence, mean values of GC were computed. For each of the *n *=* *16 sessions, in each direction, the largest *k* (out of 36) TC values were retained and compared with the corresponding GC ones. Their similarity was quantified by computing Pearson correlation coefficients between the two *k *×* n* matrices. Asterisks indicate significant correlation values (Student’s *t* test, *p < 0.05, **p < 0.01, ***p < 0.001). ***C***, Comparison of TC with CCFs. The same procedure described above for GC was performed for different values of the CCF time lag. In CCF, the frequency band leading in time is considered as the driver of the interaction. Points on the upper part of the plots denote significance of the respective correlation value (Student’s *t* test, *p* < 0.05).

10.1523/ENEURO.0484-20.2021.f2-1 Extended Data Figure 2-1 The influence of the aversive stimulus on TC during post-run and pre-run epochs. Mean TC values (n = 17 sessions, 3 rats) were calculated separately for time points from two behavioral epochs, namely, the pre-run or post-run phases, and the two running directions. All sessions have at least 20 s of total time in each of the four different data subsets shown. The plot layout and interpretation are the same as in Figure 2A (see main text for details). The characterization of a trajectory, as either safe or aversive, depends on the direction where the air puff was delivered in the run period that preceded the epoch under examination. This means that, for example, in a given pre-run phase, we consider the safe and aversive directions of the run epoch of the preceding session. Download Figure 2-1, EPS file.

10.1523/ENEURO.0484-20.2021.f2-2 Extended Data Figure 2-2 Phase differences between hippocampus and BLA for δ and θ bands. Phases were extracted from the continuous wavelet transform of the LFPs performed with analytic Morlet wavelets (cwt.m in Matlab toolbox Wavelet) in the δ (0.5–4 Hz) and θ (4–12 Hz) bands of both regions. Circular means across the electrodes in each anatomical area were computed, and unsigned phase differences were extracted from heterogenous time windows. “Wake” refers to periods in pre-sleep and post-sleep epochs where the animal is awake. The histograms show data accumulated from six sessions of Rat2. The high similarity across sessions (separate colors, stacked bars) supports that these data are robust. Download Figure 2-2, EPS file.

### Granger causality (GC) and cross-correlation functions (CCFs)

GC values were evaluated for the same set of time series used for the TCs by means of the MATLAB toolbox MVGC (Barnett and Seth, 2014). A variable *x* is called “Granger cause” for *y* if the predictability of a (chosen) model of *y*(*t*) is improved by the inclusion of the history of *x* with respect to the knowledge of the history of *y* alone. GC values are computed from the ratio of the prediction error of the full (history of *x* and *y*) and restricted (history of *y* alone) models, respectively. The original and most common formulation adopts an autoregressive model; this is followed here as well (but see Discussion, Methodological and theoretical insights). In order to deal with non-stationarities and to allow a comparison with TCs, whose embedding length typically was in the range of a few seconds, GCs were calculated in sliding windows of 10-s length and with a step size of 1 s. A specific sleep phase, NREM or REM, was assigned to each value if its relative number of time frames in the integration window was larger than 50% of the window size. The Bayesian information criterion was chosen to set the model order. Following regression and the calculation of the GC values, *p* values were obtained from the theoretical asymptotic *F* distribution (Barnett and Seth, 2014).

Pearson CCFs were computed in sliding windows of 2.5-s width and a minimum step size (50 ms). In order to test significance, for each BLA band power the related time series was divided into blocks of a size equal to its autocorrelation length. This was defined as the lag time needed to dip below an autocorrelation value of 1/*e*. The blocks were permuted, the CCFs were recalculated, and the whole procedure was repeated 200 times; *p* values were extracted by comparing the null distributions thus obtained with the true values. Block permutation was applied to preserve the autocorrelation structure of the signal while disrupting the coordination with the other signals involved (Efron and Tibshirani, 1994; Chernick and LaBudde, 2014). The same technique was used in the analysis for other statistical tests involving time series, see above, Alignment settings and Extended Data Figures 6-1, 6-2.

As for TCs, for both GC and CCF values, the significance threshold was set to 0.05. The Benjamini–Yekutieli correction was applied, and values that were not statistically significant were zeroed explicitly.

### Combined dataset of LFPs and TCs

To combine the data, we convoluted the 72 TCs (36 in each direction) with an Epanechnikov kernel with 100-ms bandwidth. The 12 band powers from hippocampus and BLA were z-scored within each session. This allowed us to meaningfully bind the TCs and LFP band powers together in an *N *×* *84 matrix where *N* is the number of time frames in all of the five behavioral epochs (pre-run, pre-sleep, run, post-sleep, and post-run) of the available sessions (see above, Experimental design and behavior). Eventually, to eliminate redundancies across bands and across TC values, we performed a PCA. We selected a number of principal components (18) in proximity to the elbow point of the explained variance (Extended Data Fig. 4-8). When comparing to a dataset without the TCs, we used only the 12 band powers and no dimensionality reduction.

An alternative to the preprocessing of the combined dataset of TCs and band powers is offered by the adoption of locally adaptive weights (LAWs; Blöchliger et al., 2015). These weights are meant to correct for the fact that different time series may be of heterogeneous importance when the system is in different states. Briefly, at each time step we count the number of transitions across the global mean (per series) in a 5-s window centered at that point. The local value is then scaled by a quantity inversely proportional to this count such that time series that are mostly noisy get lower weights and are outweighed by those showing an effective metastable dynamics. It is important to note that the weights only modulate the importance of differences in time series. Thus, if there are no differences, the weights are irrelevant as, for example, for comparisons of different points where the TCs were are all zeroed explicitly. We then performed a PCA on the data matrix after scaling it by the LAWs and kept the first five components with the largest variances. When using the dataset without TCs, we applied the same procedure but only on the 12 band powers. This included PCA.

To test how much information the TCs provide relative to band powers, we performed two types of TC permutation tests. These permutations were applied within each session, i.e., before concatenation of all the sessions. In the first test, we shuffled only the time blocks containing TCs. This preserves the relative values of TCs for different band combinations but reassigns time in a blockwise manner. In the second, the TCs were permuted between each other within the same time frame. This preserves time but redistributes the 72 values to random power band combinations.

### Progress index (PI) and Sapphire plot

The PI analysis was applied to the dataset obtained from the preprocessing procedure described above (see Combined datasets of LFPs and TCs). For a full description of the method, we refer the reader to the literature (Blöchliger et al., 2013, 2014) and for a schematic illustration to Extended Data Figure 4-1*A*. Briefly, the method rearranges the time frames (snapshots) into a new order, called PI. In the PI, neighboring points are structurally similar in the feature space. To accomplish this, an initial snapshot is chosen a priori (PI = 1). The snapshot to assign as PI = *n* (for *n* > 1) is the closest to any member of the set of the already indexed *n* – 1 snapshots. The algorithm continues progressively until all snapshots have been indexed. The addition rule corresponds to a single-linkage criterion, and the (dis)similarity we employ is the Euclidean distance in the chosen feature space. In practice, the algorithm can be cast as the task of constructing the minimum spanning tree (MST), and the PI can be viewed as a particular order of the edges of the MST. Given the size of the datasets under investigation (∼3 × 10^6^ snapshots), we here resort to the approximate version of the MST, called short spanning tree (SST), as introduced in Blöchliger et al. (2013), which offers near-linear scalability for the construction of the PI with respect to dataset size. Its construction relies on a hierarchical clustering technique (Vitalis and Caflisch, 2012), architecturally similar to the Balanced Iterative Reducing and Clustering using Hierarchies (BIRCH) algorithm (Zhang et al., 1996), whose parameters were tuned automatically according to *ad hoc* criteria. The initial PI snapshot (PI = 1) is chosen as the center point of the largest cluster found by this preparatory partitioning. The PI sequence is then plotted against different labels and annotations in the so-called States And Pathways Projected with HIgh REsolution (Sapphire) plot. A simple and helpful feature that can be plotted is the original time index (the little dots in the “Time” annotation). The second annotation to be derived purely from the time series is the “Kinetic” annotation, which measures the kinetic separation between regions to the left and to the right. In practice, given a snapshot with PI = *n*, we call *A* the set of points that lies, along the PI, within a window of length *L* centered on *n*. We then count how many transitions along the time sequence happen between the left and right halves of *A*. In other words, focusing only on the snapshots within the interval *A*, we count how many times the time sequence jumps from the set of snapshots in the right half of *A* to that in the left half and vice versa. The kinetic annotation (panel in the bottom) is proportional to the logarithm of the inverse of this count, also called cut function, and, thus, it assumes lower values when the snapshot *n* lies close to the center of a cluster, and, conversely, higher ones when *n* is located in a boundary region between different states. The window length *L* was set to 10% of dataset size throughout the analysis. Only for visualization purposes, the kinetic annotation in the Sapphire plots has been processed with a monotonic function to stress lower values and re-scaled between 0 and 1.

The PI algorithm is implemented in the software CAMPARI (http://campari.sourceforge.net/). A wrapper of the original Fortran code has been used in the analysis (R package CampaRi). This is available from a public GitLab repository (https://gitlab.com/CaflischLab/CampaRi).

### Clustering from the Sapphire plot

We implemented a processing pipeline to transform the Sapphire plot into a time-based clustering algorithm, called Sapphire-based clustering (SbC), which is designed to extract clusters by partitioning the PI sequence into consecutive chunks. A detailed description, along with applications, is provided in Cocina et al. (2020) (but see Extended Data Fig. 4-1 for a schematic). A conceptually similar approach has been used in Garolini et al. (2019) for an identification of network states.

In essence, the SbC algorithm relies on both the kinetic and temporal annotations (the two panels in the bottom of the Sapphire plot), which are first analyzed independently. For the kinetic annotation, we apply a simple peak identification algorithm to place candidate partitions along the PI. For the temporal annotation, we construct a 2D histogram (time vs PI) and parse it to identify horizontal stretches of consecutive bins with significant occupancies. Each of these represents an individual visit of a putative state in the original time series. Next, we extract a second set of putative partitions into clusters from the distribution of the boundaries of these stretches along the PI. This second set is pruned by a statistical test, which employs the Hellinger distance between the temporal distributions of adjacent stretches. The two sets of partitions, i.e., those derived from the kinetic and temporal annotations, respectively, are then merged (to eliminate redundancies), and the final trajectory clustering is delivered from this combined set.

The bin sizes in the 2D histograms on the PI axis, *w_PI_*, and on the time axis, *w_t_*, are the relevant parameters of the algorithm presented above (Extended Data Fig. 4-1*B*, panel 2). *w_PI_* sets the minimum size, and the resolution, of the clusters that we want to identify. On the other hand, *w_t_* should be of the order of the average residence time in the putative set of states. Overall, it must be recalled that the number of points in the temporal annotation is simply equal to the number of snapshots (time frames). Therefore, any combination of *w_PI_* and *w_t_* should be chosen such that the average density per bin is not too low where the threshold is set to be proportional to $w_{PI}w_{t}$. During the analysis, as a compromise between the above criteria and the final number of clusters, we chose *w_PI_* = 300 s and *w_t_* = 150 s.

The SbC result is also used, in turn, for improving the Sapphire plot itself. This is an extension to the original method as presented in Blöchliger et al. (2013, 2014). We compute a distance matrix between the clusters by taking the Hellinger distance mentioned before as a (pseudo)metric. This matrix is processed by a seriation algorithm to obtain a reordering of the clusters according to their reciprocal similarities (*seriate* in R package seriation; Hahsler et al., 2008; Behrisch et al., 2016). Finally, the PI chunks, i.e., the PI-continuous sets of snapshots representing the clusters, are reordered according to this new sequence by maintaining their internal arrangements, and the kinetic annotation is recomputed. All the Sapphire plots shown in this work were postprocessed in this way.

### Other clustering methods

Other clustering methods, which are commonly used for large datasets, were applied here for comparison. Specifically, we chose k-means clustering with mini batches (*MiniBatchKmeans* in the R package ClusterR) and k-medoids with either Euclidean or Manhattan distances (Aggarwal et al., 2001; *clara* in R package cluster). The number of clusters was always set equal to the one found by SbC for the dataset under investigation.

### Affinity scores

Each cluster is given a set of affinity scores related to labels that describe the animal’s state or behavior. For discrete variables, such as behavioral epochs (e.g., pre-sleep or run), sleep phases (i.e., NREM, REM, or wake), and the presence of SWRs, the number of occurrences of the label divided by the cluster size yielded the affinity score. For speed and firing rates, we made use of the average value within the cluster (ignoring any missing values).

The PreSleep and PostSleep labels generally refer to the periods where the rat is not located on the track and thus include awake phases. The Sleep label refers to both pre-sleep and post-sleep epochs but is restricted to time points annotated explicitly as either REM or NREM sleep phases; the remaining labels are equivalent to those shown in the Sapphire plot.

### Matching of states across rats

The clusters identified by SbC and the affinity scores related to these form the basis of a procedure that helps comparing Sapphire plots across animals. In particular, we seek the clusters of Rat1 and Rat2 that are likely equivalent to the four recognizable basins (or coarse states) of Rat3. The similarity criterion adopted takes advantage of the affinity scores. The actual composition of the four basins in terms of SbC clusters (Extended Data Fig. 4-4, 93 clusters) was determined manually but straightforwardly by using the peaks of the kinetic annotation as guidance.

The affinity scores are contained in a matrix where the rows represent the clusters and the columns the different external labels. To account for differences in scale between the different labels but also across rats, we initially rank-transform the affinity scores across the clusters (i.e., along the rows) for each rat. For simplicity, we describe the procedure using Rat1 as the example (and the four basins of Rat3 as reference). We use the affinity scores to calculate the Pearson cross-correlation matrices between the clusters of Rat1 and those of Rat3. Next, each Rat1 cluster is associated to one of the four basins of Rat3, while trying to preserve the relative size of the four coarse states. Specifically, the cross-correlation matrix is collapsed into a list of triplets composed of cross-correlation values, the corresponding Rat1 cluster, and the implied Rat3 basin, which is the one containing that particular cluster of Rat3. This list is then sorted in descending order of correlation values and, progressively, each Rat1 cluster is assigned to the corresponding basin. If the relative size of the growing basin exceeds that size as seen for Rat3, or if the given cluster for Rat1 has already been assigned, the triplet is ignored, and no association is done. By performing this procedure for the entire list, all clusters of Rat1 are eventually assigned to one of the four basins.

### Unfolding projection

To visualize the results contained in the affinity scores in two dimensions, we resort to a multidimensional scaling (MDS) technique applicable to a generic rectangular matrix *δ_ij_* with dimension *n *×* m* (Cox and Cox, 2000; Borg et al., 2018). The entries of the matrix are interpreted as the ranks that *n* judges give to *m* objects. The lower the rank the closer an object is to that judge’s taste. The unfolding method consists in projecting both the judges and the objects onto a low-dimensional space. The projection tries to ensure that objects will be found closer to those judges who have rewarded them with an optimal rank. The low-dimensional configuration is found by minimizing Kruskal’s stress function

(2) $$\sigma =\sum_{i=1}^{n}\sum_{j=1}^{m}(f(\delta_{ij})-d_{ij}{)}^{2},$$

where *d_ij_* indicates the new set of distances in the projected space, and *f(x)* is a monotonic function. In order to remove dependencies on the size of the distance matrix and on the absolute magnitudes of its elements, the actual stress values shown in the analysis are normalized as follows:

(3) $$\sigma =\sqrt{\frac{\sum_{i=1}^{n}\sum_{j=1}^{m}(f(\delta_{ij})-d_{ij}{)}^{2}}{\sum_{i=1}^{n}\sum_{j=1}^{m}f{\left(\delta_{ij}\right)}^{2}}}.$$

We used the R package smacof for the analysis (de Leeuw and Mair, 2009; Borg et al., 2018). This type of unfolding was applied to the rectangular matrices of affinity scores (see above, Affinity scores, and Fig. 5*A*, panel 3). The judges are the labels, representing, e.g., NREM, REM, PostSleep, PreSleep, SWRs, and the objects are the clusters. For this, all affinity scores were converted into row(judge)-wise ranks and, then, projected onto a 2D space (MDS coordinates). We used a ratio transformation of distances (*f(x)* = *ax*) and minimized the stress function (eq. 2) along with a penalty term. In order to check the robustness of the stress values, jackknife testing was performed by removing, in turn, a label (row) or a cluster (column) from the matrix of affinity scores.

The collective projection of all three rats is obtained by concatenating the rank-transformed affinity matrices of all the animals and performing the unfolding with the same settings presented before. Mean TCs were computed per cluster. Subsequently, we grouped TCs according to the leading band and averaged them within each group. For instance, we selected all the TCs driven by the BLA θ band, i.e., six values, and averaged them. Each of the obtained values constitutes a third dimension (TC) along with the two unfolding dimensions (MDS-1, MDS-2). In order to check whether the TC values are systematically distributed along the clusters, a plane was fitted in the [MDS-1, MDS-2, TC] space. The magnitude of the slope with respect to the [MDS-1, MDS-2] plane was extracted along with the orientation of the plane. These values can be represented by an arrow in the [MDS-1, MDS-2] space obtaining the so-called biplot. The angles indicate the directions toward which the various TCs increase the most. The lengths of the arrows are proportional to the slope and quantify how well the TC values are mapped along the indicated direction. We used a permutation test to assess the significance of the value of the slope (same as in Kyriazi et al., 2018): TC values across clusters were permuted 1000 times, and a null distribution of absolute slope values was calculated. Only TCs for which the slope exceeded the 95% quantile of the null distribution were retained for the biplot.

### Statistical analysis

All statistical tests were two-tailed tests unless stated otherwise. All tests comparing two sets of data were paired and non-parametric (Wilcoxon signed-rank test). Group data are reported with mean ± SEM, unless stated otherwise. In cases of multiple hypothesis testing, we applied the false discovery rate correction according to Benjamini–Yekutieli to the individual tests’ significance thresholds.

### Code and software accessibility

Software used to carry out the analyses is mentioned throughout the text and is available online. Customized code and scripts supporting the current study are available at https://gitlab.com/CaflischLab/unsupervised_hpc-bla.

## Results

To describe the LFP activity in the hippocampus and in the BLA (and subsequently to infer their reciprocal interactions), we used as time series the band powers computed with spectrograms on 2-s sliding windows with a 50-ms time step. We distinguish six different bands of physiological relevance: δ (0.5–4 Hz), θ (7–14 Hz in hippocampus, 4–12 Hz in BLA), β (15–30 Hz in hippocampus, 12–30 Hz in BLA), low-γ (30–70 Hz), high-γ (70–120 Hz), and, eventually, ripples and fast, respectively, for hippocampus and BLA (both 120–250 Hz; see Fig. 1*D*; Buzsáki and Draguhn, 2004; Girardeau and Zugaro, 2011; Colgin, 2016; Bocchio et al., 2017).

We have chosen the band powers to bridge distant frequency bands since they average the fluctuations in the signal across a shared time window. In this way, we homogenize the intrinsically heterogeneous timescales of variations in the LFPs across the different bands. Possible drawbacks of our approach are addressed in the Discussion section.

### Interaction analysis between behavioral epochs and sleep phases

We use TC as a measure of directed interactions between the hippocampus and the BLA (Harnack et al., 2017). The method is based on a time-lagged embedding procedure derived from an underlying theory in the field of dynamical systems and nonlinear time series analysis (Kantz and Schreiber, 2004). Note that the term “causality” has a different notion in that field, and it is not directly related to the causality assessment exerting neural manipulations (more in Discussion). We provide here a conceptual explanation of the methods, and we refer the reader to the Materials and Methods section and to the original article for more details (Harnack et al., 2017). The time-lagged embedding consists in generating from a time series *x*(*t*) a space of higher dimensionality *m*, called *X*, where the additional dimension corresponds simply to values of *x* delayed by multiples of a time lag *τ*; in explicit terms, X(t)={x(t),x(t−τ),...,x(t−(m−1)τ)}. Takens’ theorem (Takens, 1981) stipulates that a lagged embedding of sufficient dimensionality is able to reconstruct the manifold generated by the underlying dynamical system (Kantz and Schreiber, 2004; Bradley and Kantz, 2015). In TC and our application of it, this implies that from the embedding of the hippocampal and BLA time series we should be able to extract relevant information regarding the reciprocal coupling of the two quantities. In particular, it should be possible to quantify to which degree the information of a time series is determined by the history of the other. A practical explanation is illustrated in Figure 1*C*. In the first example (upper panels) a case of unidirectional coupling is shown: *x* influences *y*, that is, the time evolution of *y* depends on *x* but not vice versa. In the embedded space *X*, neighboring points of *X*(*t*) are identified (blue circle) and the counterparts in *Y*, i.e., those with the same time indexes, are mapped graphically (straight lines from left to right). Since *x* is fed no information from *y* in this example, the points projected in *Y* will be poorly “clustered” (i.e., the projected neighborhood diverges). When considering the other direction and, thus, mapping the neighborhood of *Y*(*t*) (red circle) onto *X*, the projected points in *X* will lie closer to each other since the evolution of *y*(*t*) depended on *x*. In the second case (lower panel), *x* and *y* are reciprocally coupled, and both projected neighborhoods span compact areas. Generally speaking, the effective sizes of these neighborhoods will depend on the effective degree of influence of one time series onto the other. TC quantifies these as expansions, making it possible to derive a measure for the asymmetries in reciprocal influence between the two time series.

To be able to isolate significant directed interactions, the TC value at each time step is compared with the chance level distribution, which is obtained by mapping the variable’s neighborhood randomly. TC values that do not pass this significance threshold are zeroed explicitly (*p* > 0.05, Benjamini–Yekutieli corrected; see Materials and Methods, Topological causality). Values of TC are computed between the band powers in the hippocampus and BLA (36 pairs, two directions). Importantly, despite the reliance on time windows for both spectrograms and manifold reconstruction (2 s and 2 min, respectively), the resultant signals are able to resolve fast processes on the subsecond time scale, see Figure 1*E*. In the following, when discussing TC results, we will mostly use the terms “influence” or, more generically, “interactions” without referencing causality. We confront this terminological issue in detail in the Discussion section.

#### Pronounced interactions between hippocampus and amygdala occur with characteristic patterns during both NREM and REM sleep yet differ less between post-sleep and pre-sleep than between post-run and pre-run phases.

The mean values of TC during the sleep epochs are shown in Figure 2*A*. The distinction between NREM and REM periods has been derived from the ratio between hippocampal δ and θ band powers (see Materials and Methods, Experimental design and behavior). During NREM, reciprocal influences between the low-frequency bands are prominent, in particular for the hippocampal δ, θ, and β bands, as well as for the δ and θ bands in the BLA. To a lesser degree, δ and θ bands in the BLA appear to be driven by the hippocampal ripples band more than by the γ bands. In addition, we observe that the BLA fast band is driven by hippocampal activity across the entire frequency range. During REM, some of the aforementioned interaction patterns decrease considerably, in particular those concerning the hippocampal β band and those involving the low frequencies of the two regions (δ and θ). A notable exception to this latter statement concerns the hippocampal, θ-driven TCs, which are prominent and match the expected characteristics of REM sleep. These TCs are strong relative to most of the BLA frequencies and are complemented by BLA-driven TCs on the two highest frequency bands. The TCs found for the hippocampal θ band are mirrored partially in the high-γ band. Based on the BLA-driven TCs in the high-frequency bands, there does appear to be some reciprocal communication during REM. The differences between post-sleep and pre-sleep epochs (Fig. 2*A*, right panel) are generally much lower than those between sleep phases (Fig. 2*A*, bottom panel; Wilcoxon signed-rank tests, *n *=* *16). One exception to this regards the β band of the BLA where two TC differences are significant. This contrasts sharply with the analysis performed on pre-run and post-run epochs. Here, a marked enhancement of TC values is visible during the post-run epoch (Extended Data Fig. 2-1; Wilcoxon signed-rank tests, *n *=* *17). This difference is prominent for most frequency bands and in both directions. Changes in the influence exerted by the hippocampal θ, β, high-γ, and ripples bands are pronounced in the safe running direction but somewhat weakened, for the θ and ripples bands, in the aversive direction. Conversely, interactions exerted by the BLA on the hippocampus are strengthened in post-run epochs in the aversive direction: δ-driven and, especially, θ-driven interactions are particularly enhanced. We stress that the aversive stimulus is absent in the post-run epoch, so the differences in Extended Data Figure 2-1 are likely related to the memory retrieval of the previous conditioning experience.

Apparent interactions between brain regions can also result from volume conduction, both incoming from external regions or, specifically, from hippocampus to BLA. This mechanism might contribute to any signals suggesting interactions involving low-frequency bands like δ and θ (Łe ¸ski et al., 2013; Bertone-Cueto et al., 2020). Regarding the θ band, however, several pieces of evidence of both behavioral and biological nature suggest that volume conduction is unlikely to be a major factor; these are summarized in Pape and Pare (2010) and Bocchio et al. (2017). For example, it has been observed that activities in the θ band of the lateral amygdalar and CA1 hippocampal regions are synchronized during fear memory retrieval but not during exploratory behavior (Narayanan et al., 2007; Seidenbecher et al., 2003). To estimate the potential influence of volume conduction on our analysis, Extended Data Figure 2-2 shows computed phase differences for LFPs filtered to the δ and θ bands between the two anatomical areas in different time epochs. While phase differences close to zero would correspond to expectation for volume-conducted oscillations (Nolte et al., 2004), the histograms are generally broad and not consistently peaked at zero phase difference for the θ band. This corroborates the aforementioned hypothesis that volume conduction plays a negligible role in this band. For the δ band, phase differences do consistently show the highest values in the proximity of zero in Extended Data Figure 2-2. While this does not need to result from volume conduction, the data in conjunction with literature observations of volume conduction in this frequency band in nearby regions (Bertone-Cueto et al., 2020) suggest a more cautious interpretation for δ-δ interactions.

#### Two independent measures correlate with TC if the memory lengths are matched.

To assess the robustness of our analysis, we next compared TC with two common linear techniques used to investigate directed interactions between oscillatory activities and connectivity between distant brain regions. Specifically, we chose GC and CCFs with varying time lag. We restricted ourselves to the same four data subsets shown in Figure 2*A*. GC and CCF values were computed for all the 36 frequency band combinations. This was done separately for both directions of influence. For each session and time period, we selected the largest 6, 18, or 36 (i.e., all) TC values and calculated their Pearson correlation with the respective GC and CCF values. GC and TC are compared in Figure 2*B*, which highlights that there are significant levels of (linear) correlation for the data from the NREM phase. These tend to be generally lower for post-sleep with respect to pre-sleep, in particular when selecting a lower number of frequency band combinations. In REM, GC and TC values are generally uncorrelated for the BLA → hippocampus direction, and, as for NREM, pre-sleep patterns display higher correlations than post-sleep ones. In Figure 2*C*, the comparison of TC values with CCF values with time lags of up to ∼3 s is presented. The emerging picture is very similar to that seen for the TC/GC comparison if short time lags of <1 s are considered. For data taken from REM sleep, correlations of BLA-driven interactions are slightly more erratic as seen from the dependence on the number of variable pairs considered and the lower significance of the results (circles on top). Moreover, TC and CCF values start to anti-correlate almost everywhere when the time lag exceeds a threshold of ∼1–1.5 s. This value is comparable to the average embedding length determined for TCs, which depends on both a base lag and a choice of dimensionality (see Materials and Methods, Topological causality). Why do TC values correlate only weakly or erratically with GC/CCF values if the source data are restricted to the REM period? First, it is important to point out that REM sleep is characterized by shorter episodes than NREM. In this case, the assignment of the REM label to the computed value, either TC, GC, or CCF, can be ambiguous given that each technique has its own different integration window that may extend across adjacent NREM time frames. Second, the TCs in the BLA → hippocampus direction during REM are confined to the fast frequency bands, and the underlying activities might be too short-lived to be picked up by a correlation measure. Third, both classical GC (as used here) and CCF are intrinsically linear techniques whose application to nonlinear systems may lead to equivocal or misleading results (Sugihara et al., 2012; Harnack et al., 2017; see Discussion, Methodological and theoretical insights).

#### SWRs are associated with unidirectional communication from the hippocampus to the amygdala during NREM sleep and awake phases.

**Figure 3.**
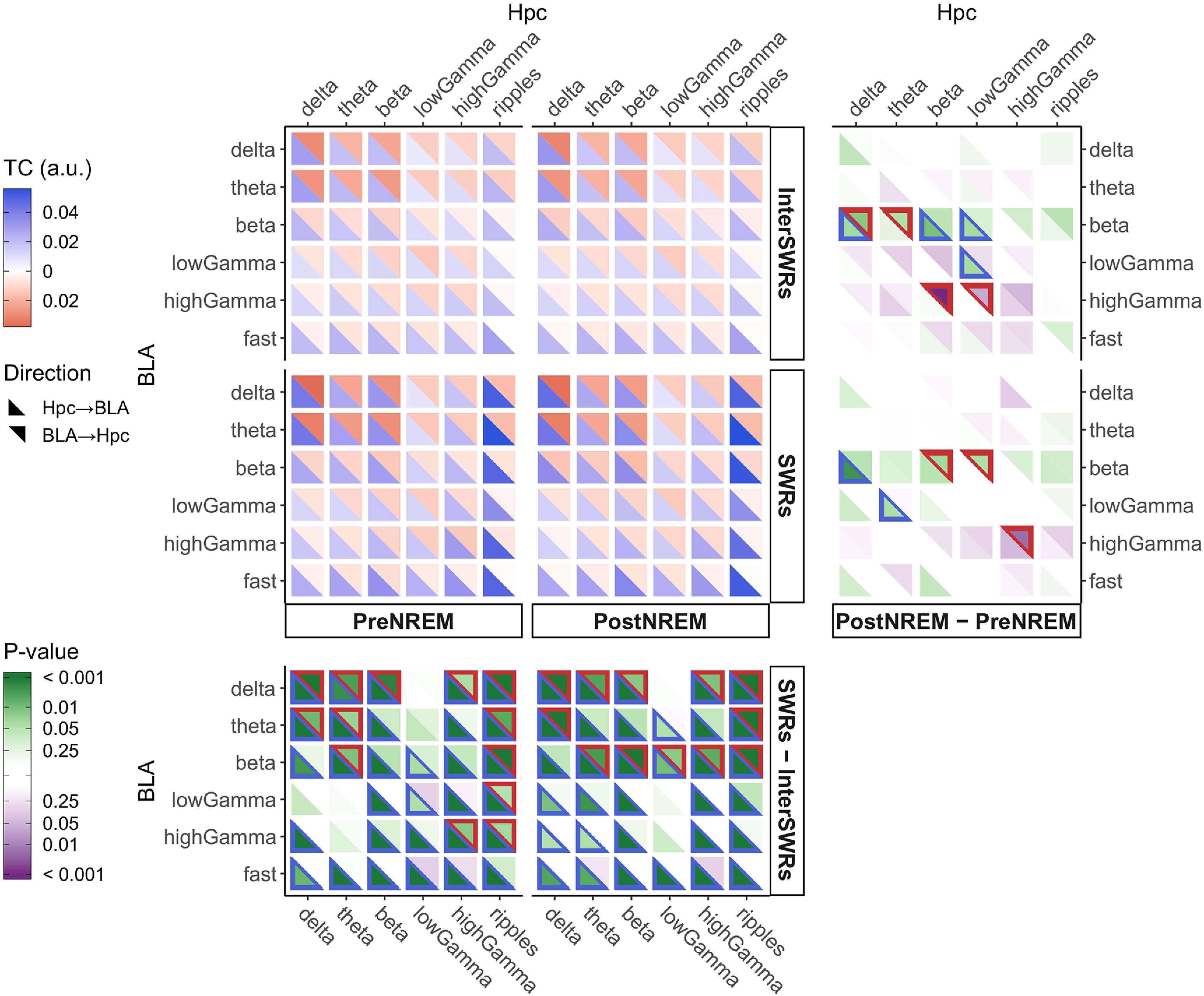
TC analysis during NREM sleep. Following the same procedure as for Figure 2*A*, we display TC results for pre-NREM and post-NREM sleep in the presence or absence of SWRs (*n *=* *20, 3 rats). All sessions included show at least 5 s of total time featuring SWRs in both the pre-NREM and post-NREM epochs. The analogous figures for the wake phases and for the run epochs are shown in Extended Data Figures 3-1, 3-2, respectively. TC differences are robust to the choice of alignment between TCs and SWRs timestamps (Extended Data FIgures 3-3, 3-4). See details in Materials and Methods, Alignment settings.

Because of the results shown in Figure 2*B*,*C*, we focus for the following analysis only on NREM sleep and on the potential role of SWRs for the modulation of interaction patterns. To answer this question, we partitioned the NREM data into subsets featuring SWRs and those that do not (inter-SWRs; see Materials and Methods, Experimental design and behavior for the procedure used to detect SWRs). From Figure 3, we can glean that there is a broad influence of the hippocampus on the BLA driven by its ripples band and, to a lesser extent, by its low-frequency bands, including δ, θ, and β. While these hippocampal drives are enhanced during SWR phases, the reciprocal BLA-driven interactions seem to not be affected strongly by the SWRs (Wilcoxon signed-rank tests, *n *=* *20). This suggests the interpretation that SWRs either facilitate or are at least a hallmark of communication going out from the hippocampus. Interestingly, although SWR phases show higher TC values overall for hippocampus → BLA, significant differences between pre-sleep and post-sleep phases are more commonly found for data from time periods devoid of SWRs.

10.1523/ENEURO.0484-20.2021.f3-1 Extended Data Figure 3-1 TC analysis for awake phases. From the data corresponding to awake phases, mean TC values (n = 20 sessions, 3 rats) were calculated separately for the four combinations of the two behavioral epochs, pre or post, and the presence or absence of SWRs. All sessions show at least 5 s of time featuring SWRs in both pre-epoch and post-epoch. The plot layout and interpretation are the same as that in Figure 2A (see main text for details). Download Figure 3-1, EPS file.

10.1523/ENEURO.0484-20.2021.f3-2 Extended Data Figure 3-2 Occurrence of SWRs during the run phases. We calculated mean TC values (n = 13 sessions, 3 rats) separately for the four cases resulting from the combinations of the labels safe/aversive direction and presence/absence of SWRs during the run epoch. All sessions show at least 2 s of time featuring SWRs: this holds independently for both directions. The plot layout and interpretation are the same as in Figure 2A (see main text for details). Download Figure 3-2, EPS file.

10.1523/ENEURO.0484-20.2021.f3-3 Extended Data Figure 3-3 Variation of TC absolute differences with respect to time lag during NREM sleep. For each lag τ on the x-axis, TC values are shifted forward (τ > 0) or backward (τ < 0) in time (see Materials and Methods, Alignment settings between TC, band powers, and external labels for details). The overall differences between the different sleep/SWRs phases were computed based on values similar to those used to perform the Wilcoxon signed-rank test in Figure 3, right and bottom panels. For each of the combinations of sleep/SWRs phases and for both directions of influence, we show the average of the absolute values (solid lines) along with their respective standard deviations (shaded areas; n = 20 sessions, 3 rats). Dashed lines represent the average across sessions of the 95% quantiles of the chance level distributions (block permutation of time bins). When we differentiate between pre-NREM and post-NREM, the total variation, in both directions of influence, is generally higher than the underlying chance level. Thus, this derived global measure of interaction indicates a robust difference even when the individual TC differences may appear marginal. As expected, within the short time windows of SWRs, these values become sensitive to the lag of the TC, especially for hippocampus-driven interactions. In fact, if we consider the difference matrices between SWRs and inter-SWR phases (bottom panels), there is a clear regime of more or less constant differences within a window of ∼1 s. While this plateau is slightly asymmetric around the choice we made, this choice nonetheless appears reasonable. Download Figure 3-3, EPS file.

**Figure 4.**
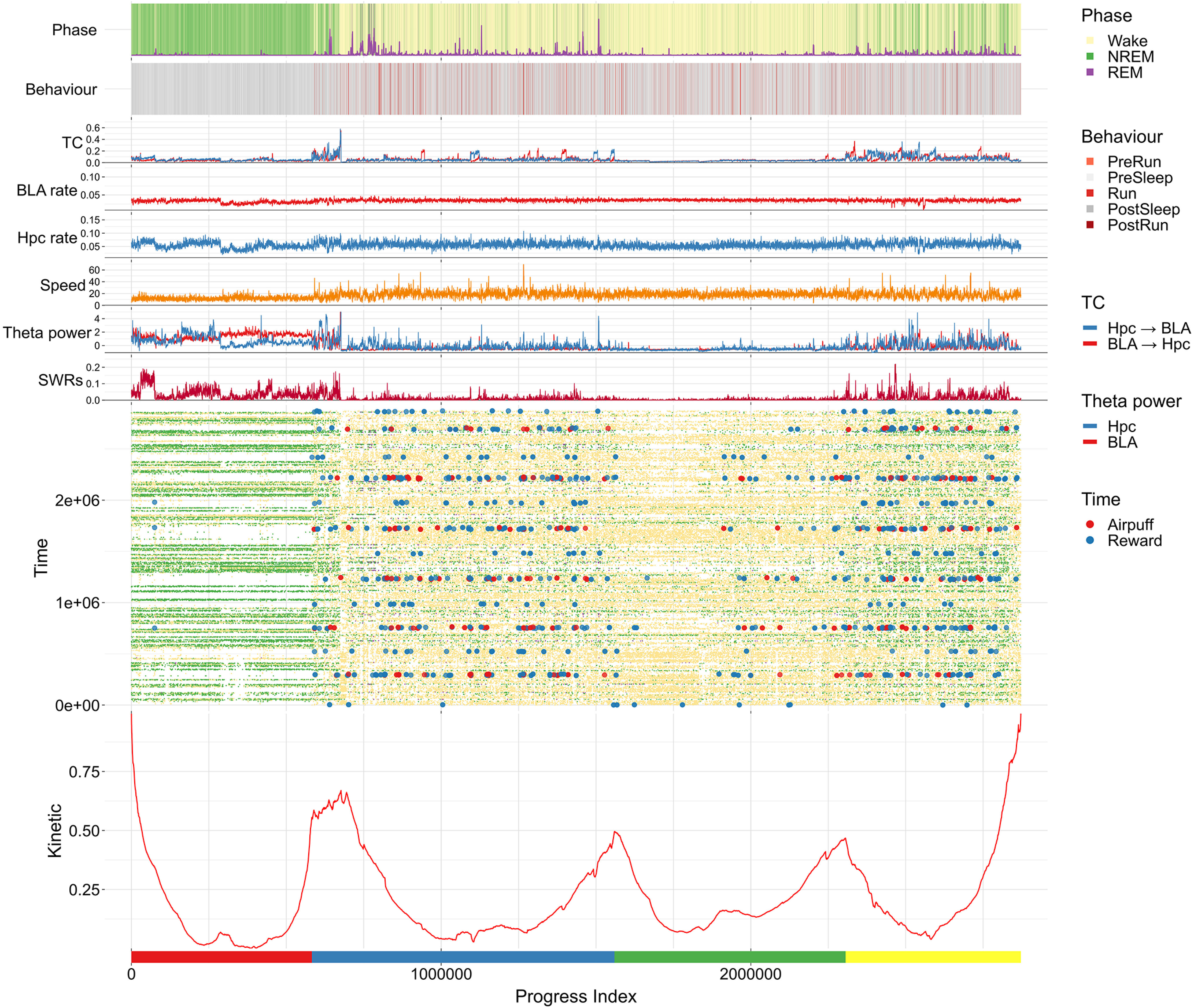
Sapphire plot of the combined dataset of band powers and TCs. We show the results for Rat3 (6 sessions; all five behavioral epochs included; ∼40 h in total; 50-ms resolution). The time series was rearranged according to a similarity criterion in the so-called PI (see Materials and Methods, Progress index (PI) and SAPPHIRE plot, and also Extended Data Fig. 4-1*A* for a schematic illustration). The kinetic annotation (bottom, red curve) highlights transitions between putative states. Directly above, the original position along the time sequence is plotted as a function of the PI. The color code is given by the sleep phase (small dots, see “Phase” legend) and by the type of events encountered while awake (circles, see “Time” legend). Because of limits to plotting resolution, a regular subsampling by a factor of ∼10 was applied along the PI dimension. In the five panels above the Time annotation, we show, respectively, the histogram of SWRs, the z-scored θ powers of both regions (thresholded at 5), movement speed, firing rates of pyramidal neurons in hippocampus and BLA, and the average TCs in both directions. These six features were smoothed with a moving average filter employing a window of 500 points along the PI before plotting. The scales on the *y*-axes of these six annotations are homogenized across all Sapphire plots. Finally, the two topmost panels indicate by color sleep phases and behavioral epochs, respectively (legends on the right). To be able to spot the REM phase more easily, the top panel is augmented with a curve indicating the fraction of REM-labeled points on a sliding window of 25 s. Note that the pre-run and post-run epochs form just ∼2% of the overall time series, thus making them difficult to distinguish in the plot. At the very bottom of the plot, colored bars indicate four coarse states. Maintaining this color scheme, a matching procedure between these coarse states and putative ones in the remaining two rats (Extended Data Figs. 4-2, 4-3) was applied (Materials and Methods, Matching of states across rats) to facilitate comparisons across animals. Extended Data Figure 4-4 shows the same Sapphire plot highlighting the clusters derived from SbC (see text and Extended Data Fig. 4-1*B*). We applied two different preprocessing pipelines to the combined dataset of TCs and band powers, which differ most strongly in the number of principal components retained, specifically, 5 and 18 (see details in Materials and Methods, Combined dataset of LFPs and TCs). The Sapphire plots presented here and in Extended Data Figures 4-2, 4-3 for the remaining rats are for the dataset with 18 dimensions, see also Extended Data Figure 4-8; the results obtained with the alternative preprocessing (5 components) are shown instead in Extended Data Figures 4-5, 4-6, 4-7.

10.1523/ENEURO.0484-20.2021.f3-4 Extended Data Figure 3-4 Variation of TC absolute differences for awake phases as a function of lag. The data were separated per behavioral epoch, pre or post, and the presence or absence of SWRs during awake periods (n = 20 session, 3 rats). The figure is analogous to Extended Data Figure 3-3, and, as in that figure, our choice of relative alignment appears reasonable by observing that the absolute differences between SWRs and inter-SWR phases are sustained around our working point (τ = 0). In addition, these variations are larger and appreciable also for the BLA → hippocampus direction. Download Figure 3-4, EPS file.

We also investigated the variation in TCs with respect to the presence of SWRs both during the wake periods in pre-sleep and post-sleep epochs (Extended Data Fig. 3-1), and during the run epoch itself (Extended Data Fig. 3-2). In the former, with respect to NREM, SWRs are associated with significantly increased interactions between BLA δ and θ bands and most bands of the hippocampus (Wilcoxon signed-rank tests, *n *=* *20). On the other hand, signal inputs from the BLA to the high-frequency bands of the hippocampus are weakened considerably. Instead, during the actual run epoch (Extended Data Fig. 3-2), the modulation by SWRs is much less pronounced overall: it appears to be restricted primarily to hippocampus-driven interactions in the ripples band (Wilcoxon signed-rank tests, *n *=* *13). Furthermore, TC values during either SWR intervals or in between do not seem to distinguish the running directions strongly. The clearest recognition appears to come from BLA-driven processes, slightly more so in the absence of SWRs. These enhancements found when the rat encounters the aversive stimulus could be related to the stimulus itself or to fear conditioning. We also investigated how the relative alignment between TC and SWR labels can affect the analysis. Results shown in Extended Data Figures 3-3, 3-4 support our adopted settings (see also Materials and Methods, Alignment settings).

### Global interaction analysis of data-derived, neural states

Similar patterns of directed influence, reciprocal or not, appear across different epochs, different sleep phases, or different behaviors. These patterns can reveal information that goes beyond the annotations usually employed to characterize the animal’s state, e.g., running speed or neuronal firing rates. We provide a methodology to assess whether TCs, along with band powers, can help to reveal putative states related to activity in the hippocampus, the BLA, and the coordination between them. For this purpose, we use, for each session, the entire time series without selecting any particular epoch of interest a priori, i.e., without discarding periods that may be deemed irrelevant. In detail, we merged the time series of TCs and the LFP band powers. The resulting joint dataset contains 84 time series: 6 power bands each for hippocampus and BLA and 36 TC values in either direction of interaction. These data had to be preprocessed to feasibly combine the two different classes of time series. The projection of the resulting dataset onto its first 18 principal components was used for further analyses (see Materials and Methods, Combined dataset of LFPs and TCs).

Our first goal is to highlight and annotate putative neural states that are visited recurrently (in time) throughout the experiment. To accomplish this, we use an unsupervised algorithm that reorders the time series as the so-called PI (Blöchliger et al., 2013). From this reordering, we obtain with the help of suitable annotations a Sapphire plot (Blöchliger et al., 2014). These scalable techniques were originally developed for the analysis of molecular dynamics simulations, but their applicability is general for time series data (Blöchliger et al., 2013), as shown also in (Klein et al., 2017). While the details can be found in the Materials and Methods section, we provide a short rationale next (see also Extended Data Fig. 4-1). A state is a collection of time points that have similar properties. Unless specified otherwise, these properties are the aforementioned 18 principal components (of the 12 band powers and 72 TC values). With respect to our data, the terms “clusters” and “states” are used interchangeably given that the latter are identified by a clustering method. The reordering into the PI attempts to group all instances of mutually similar time points, which may occur at vastly different times, into the same neighborhood along the PI. The peaks in the kinetic profile help to identify areas with few transitions between adjacent PI segments, i.e., they suggest the boundaries by which to delineate different states (Fig. 4, bottom panel). The original temporal index, along with time series and labels which may or may not be part of the original dataset, are then reordered according to the PI sequence and plotted on top of the kinetic profile. In this way, they provide further recognition of states and offer an initial characterization.

10.1523/ENEURO.0484-20.2021.f4-1 Extended Data Figure 4-1 Schematic of Sapphire methodology. A, PI construction. The first snapshot (time point) is arbitrarily selected and given PI = 1 (left panel). At any stage, the next index in sequence (violet dots with orange halos) is assigned to the snapshot that is closest to any of the points already added to the PI (orange and numbered dots). The Sapphire plot (right) is composed by plotting two annotations ordered according to the PI. On the upper panel, the original time values per snapshot are shown (gray arrows in the left panels denote the sequence of time). This represents the Time or temporal annotation. On the lower panel, the kinetic annotation, in its simplest version, indicates the kinetic distance of the group of snapshots to the left of the specific PI value from that to the right. Focusing on PI = 50, the group on the left (orange area in the Sapphire plot) is connected by only four temporal transitions to the other cluster (orange edges on the respective PI = 50 panel). In graph-theory, this is equivalent to find the minimum cut between the two subsets (dashed line). The kinetic annotation is inversely proportional to this number of connecting edges. For the lowest and for the highest values of PI (see, e.g., PI = 1), the kinetic function is necessarily large due to the small size of one of the two subsets. B, SbC. A summary of the method is provided in Materials and Methods, Clustering from the Sapphire plot, while full details can be found in Cocina et al. (2020). Below we present the key aspects. A simulation of n-butane of 105 snapshots with a sampling time of 50 fs serves to illustrate the method [molecular cartoon above (1)]. The system is described by the three dihedral angles associated with the three carbon-carbon bonds (denoted as HCCC, CCCH, CCCC). These have been used for the construction of the PI along with a proper angular metric. The resulting Sapphire plot is shown in (1). The annotation on top shows the actual dihedral angle values. The clustering method proceeds by identifying putative partitions along the PI. This is done separately in the Time (2) and in the Kinetic annotation (3). In (2), the analysis is based on an underlying 2D histogram (PI and time axes) that serves to identify densely clustered blocks of dots (gray). These correspond to individual residence periods in a given putative state, which is delimited by two partitions. An initial set of partitions (green vertical lines) is inferred from the end points of these blocks. The temporal distributions of adjacent candidate states are quantified by their Hellinger distance, which is used as a test statistic (green points in the lower panel; these are aligned to the partitions delimiting two adjacent states). A null distribution is created from the repeated shuffling of the PI values across the two adjacent states within restricted temporal windows (i.e., similar to the aforementioned blocks). The partition is then kept if the respective Hellinger distance is statistically significant (p < 0.01), otherwise it is rejected (circled dots). The kinetic annotation, see (3), is normalized by subtracting the analytical curve derived from a random exploration of the phase space (black line; see Blöchliger et al., 2013). A smoothing filter is applied (orange curve) and peaks, corresponding to the partitions, are identified (crosses). The peaks that do not satisfy specific criteria of prominence are rejected (circles). Partitions from (2) and (3) are eventually combined (green and orange lines, respectively) and merged if they overlap (black lines). Reprinted (adapted) with permission from Cocina et al. (2020). Copyright (2020) American Chemical Society. Download Figure 4-1, EPS file.

10.1523/ENEURO.0484-20.2021.f4-2 Extended Data Figure 4-2 Sapphire plot for Rat1. For the description of the labels and layout, please refer to Figure 4 in the main text. The colored bars at the bottom indicate the results of the matching procedure between the four coarse states of Rat3 (Fig. 4) and the clusters that can be derived from the Sapphire plot shown here (Rat1). As for Rat3, the clusters are extracted with the SbC method that is described in Materials and Methods, Clustering from the Sapphire plot. The matching procedure is explained in Materials and Methods, Matching of states across rats. We discarded one of the seven sessions due to its poor compatibility with all the other sessions. The raw data were the 18 principal components extracted from the 84-dimensional space of band powers and TCs [see Materials and Methods, Progress index (PI) and Sapphire plot]. The remaining sessions comprise all five behavioral epochs. Note that Rat1 not only showed a reduced rate of crossings in the run phase compared to Rat2/3 (∼0.3, ∼1.0, and ∼2.0, respectively) but also exhibited a ∼30-fold difference in this rate across sessions (∼2- to 3-fold for Rat2/3). Download Figure 4-2, EPS file.

10.1523/ENEURO.0484-20.2021.f4-3 Extended Data Figure 4-3 Sapphire plot for Rat2. For the description of the labels and layout, please refer to Figure 4 in the main text. As for Extended Data Figure 4-2, the colored bars at the bottom are used to indicate the clusters that were matched to the four coarse states identified for Rat3 [Fig. 4; see Materials and Methods, Progress index (PI) and Sapphire plot and Matching of states across rats]. All seven sessions for this rat and all five behavioral epochs were used in the analysis. The raw data were the 18 principal components extracted from the 84-dimensional space of band powers and TCs [see Materials and Methods, Progress index (PI) and Sapphire plot]. Download Figure 4-3, EPS file.

10.1523/ENEURO.0484-20.2021.f4-4 Extended Data Figure 4-4 Sapphire plot for Rat3 with highlighted clusters. This is the same as Figure 4 in the main text only that the separations between identified clusters are shown as vertical lines (see Materials and Methods, Clustering from the Sapphire plot for details). Download Figure 4-4, EPS file.

**Figure 5.**
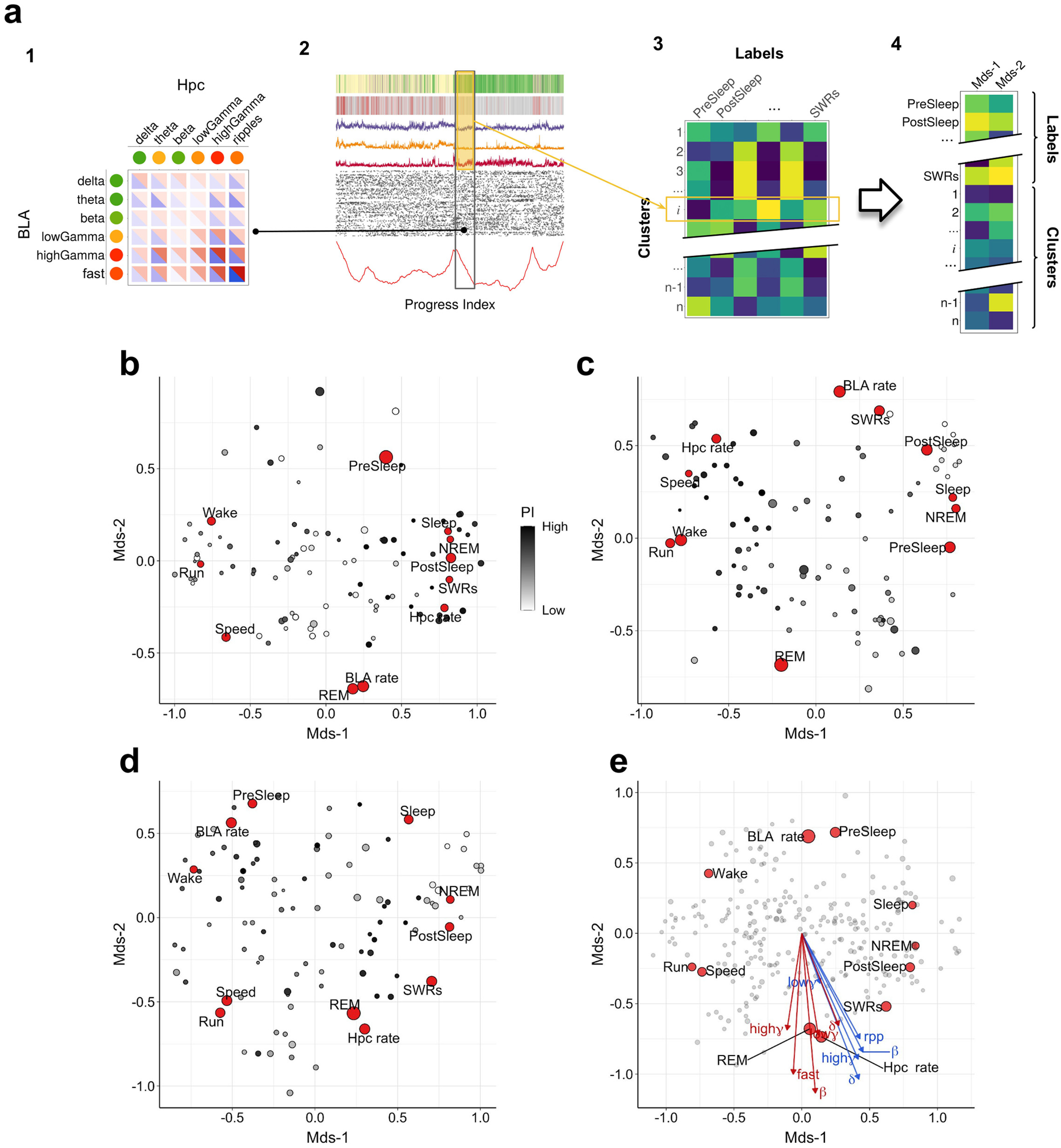
Unfolding projection of labels and clusters. ***A***, Each cluster (1) visualized by the Sapphire plot and extracted by SbC (2) represents a particular combination of band powers (higher magnitude from green to red) and TC values (same palette of Fig. 2*A*). The 11 labels (or judges, see Materials and Methods, Unfolding projection), characterizing each time frame and displayed in the Sapphire plot (2, orange box), are summarized in each cluster by their respective affinity scores (3). For each cluster, these values are computed either as fractions of occurrences for discrete labels, e.g., “PreSleep”, “NREM”, or as average values for continuous ones, e.g., “Hpc rate”, “Speed”. The affinity matrix is then used as a distance matrix to project both labels and clusters in a 2D space (4; see text for details). ***B–D***, Unfolding projections for Rat1, Rat2, and Rat3, respectively. Clusters identified by the SbC method (gray-scale dots; brightness according to their mean PI values in the corresponding Sapphire plots) and labels (red circles) are plotted in the reduced MDS space. The size of the dots is proportional to their contribution to the total stress. There are 89, 89, and 93 clusters for Rat1, Rat2, and Rat3, respectively. ***E***, Unfolding projection for all three rats and biplot of the relevant mean TC components. Here, the color of the points is not related to any variable. The TCs were averaged over the leading bands but separately per direction of influence. The arrows show the results from fitting a plane in the three-dimensional space of MDS-1, MDS-2 and a given mean TC. The direction gives the orientation of the plane, and the length is proportional to the slope. Blue arrows refer to hippocampus → BLA, red to the opposite. For the sake of visualization, Greek symbols and abbreviations of leading band names are used. Only planes with significant slopes are depicted in this way (permutation test: *n *=* *271, *p* < 0.05). See details in Materials and Methods, Unfolding projection.

10.1523/ENEURO.0484-20.2021.f4-5 Extended Data Figure 4-5 Sapphire plot for Rat1 with alternative preprocessing. Differing from Extended Data Figure 4-2, we included all six sessions, and the source data were preprocessed differently. Briefly, we computed LAWs from the 84 combined band powers and TC values and used them to strengthen the impact of temporally stable signals (see Materials and Methods, Combined dataset of LFPs and TCs for details). This is expected to be more sensitive to episodes of sustained activity in particular power bands. Only the first five principal components (by variance) of the resulting dataset were retained. As a result of this preprocessing pipeline, the partitioning of labels is much more localized compared to Extended Data Figure 4-2. This discrepancy is a particular feature of the data for Rat1, and it arises primarily because the partitioning in Extended Data Figure 4-2 is poor. Download Figure 4-5, EPS file.

**Figure 6.**
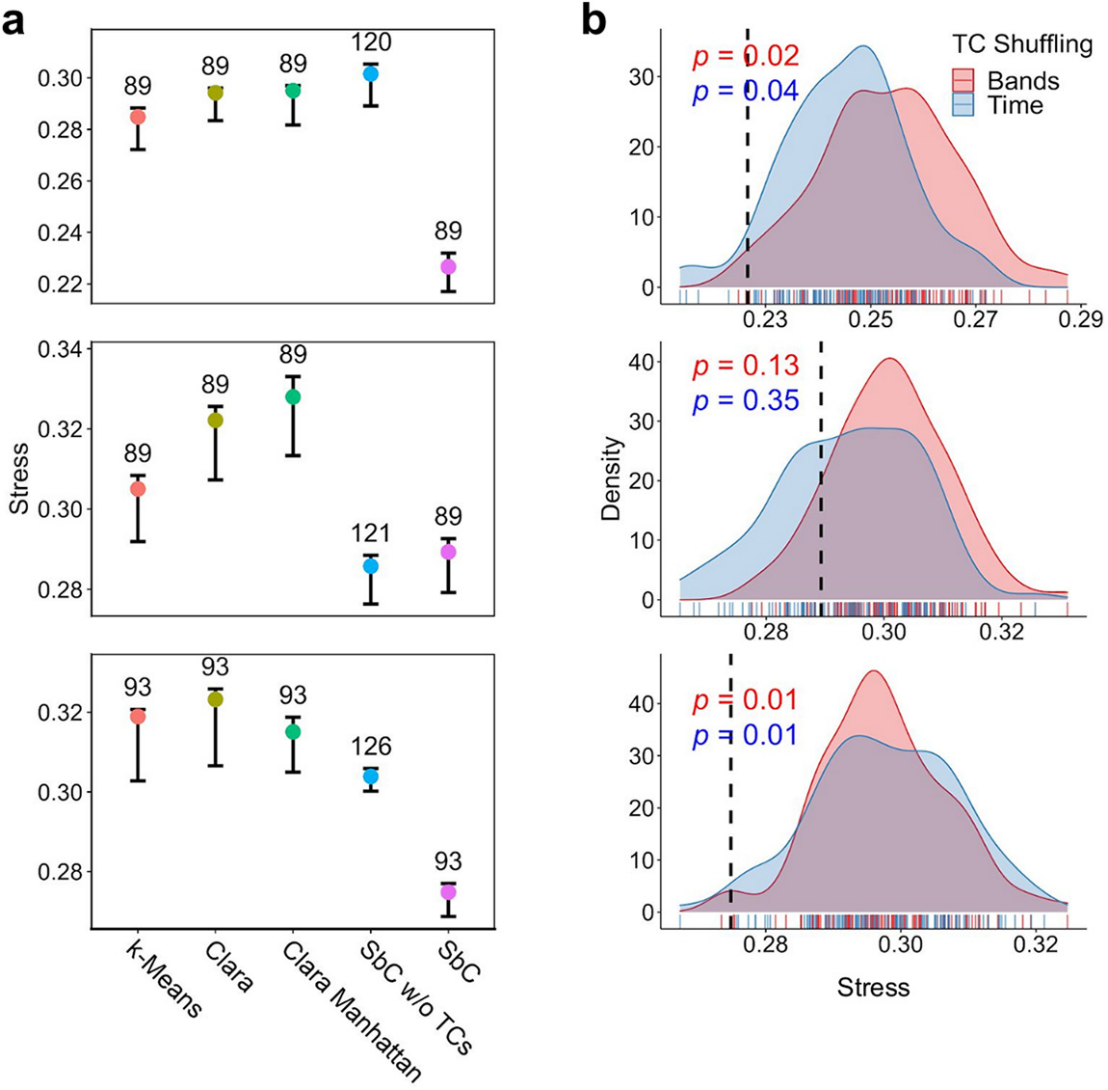
Comparison and analysis of the unfolding stress values. Top, middle, and bottom, Results for Rat1, Rat2, and Rat3, respectively. ***A***, Stress values for SbC and clustering methods used commonly for large datasets. SbC results are included with and without inclusion of TCs. Error bars represent the 95% confidence interval resulting from jackknife resampling. The numbers of clusters are indicated on top. Note that a truly random embedding produces a distribution of much larger stress values, namely, ∼0.38 for the 5th percentile (permutation of time bins after actual clustering). ***B***, Distribution of stress values after application of the SbC clustering to two different randomized datasets. In blue, the 72 TC values were jointly shuffled in blocks along time within each session (randomizing only time while maintaining the coordination between TCs within each time block). The block size was equal to the average autocorrelation length across the 72 TCs. In red, for every time point, the 72 TC values themselves were permuted (randomizes only the pairs of power bands). For each type of shuffling, 100 permutations were performed. Vertical dashed lines indicate the actual values, and *p* values are shown for each type of shuffling. The contribution of each unfolding label to the global stress value is investigated in Extended Data Figure 6-1 (see Materials and Methods). The analogous results derived from the combined dataset with alternative preprocessing are presented in Extended Data Figure 6-2.

10.1523/ENEURO.0484-20.2021.f4-6 Extended Data Figure 4-6 Sapphire plot for Rat2 with alternative preprocessing. The raw data were the same as in Extended Data Figure 4-3, and the preprocessing is the same as that described for Extended Data Figure 4-5. Note that this plot is much more similar to Extended Data Figure 4-3 than Extended Data Figure 4-5 is to Extended Data Figure 4-2. Download Figure 4-6, EPS file.

10.1523/ENEURO.0484-20.2021.f4-7 Extended Data Figure 4-7 Sapphire plot for Rat3 with alternative preprocessing. The raw data were the same as in Figure 4 in the main text, and the preprocessing is the same as that described for Extended Data Figure 4-5. Download Figure 4-7, EPS file.

10.1523/ENEURO.0484-20.2021.f4-8 Extended Data Figure 4-8 Explained variance with respect to the number of principal components. This figure refers to the dataset composed of the 12 band powers (6 each for hippocampus and amygdala) and the 72 TC values (36 per direction of influence). Download Figure 4-8, EPS file.

#### Neural states at coarse resolution isolate both sleep phases and emotional stimuli.

Results for Rat3 are shown in Figure 4, while those for the other two animals are displayed in Extended Data Figures 4-2, 4-3. Four coarse states (basins) can be recognized with ease from the combination of the different annotations (Fig. 4, colored bars at the bottom). From the kinetic annotation (bottom) and the sleep phase annotation (top), we find that most of the NREM sleep data points are found in the first large coarse state (basin) on the left. The remainder of the NREM-labeled time points appear in the transition area immediately to the right of this basin as well as in the rightmost one. Time frames from NREM sleep and awake periods are interspersed in these two areas presumably because they share bidirectional and unidirectional TC patterns (“TC” annotation). On the other hand, REM-labeled points appear in the second and, to a lesser extent, in the last basin, along with time points when the animal was awake. Consistent with Figure 2*A*, these two regions show two different degrees of TC activity, that is, lower in the second coarse basin from the left and higher in the rightmost one. Air puff and reward times (the circles in the Time annotation) are mostly scattered throughout the two large basins that display noticeable TC values. Interestingly, the SWRs (“SWRs” annotation) mirror almost exactly the same pattern as the TCs except in the leftmost NREM basin indicating an apparent decoupling of the BLA from the hippocampus during NREM sleep. A similar observation can be made for power in the θ band (“Theta power” annotation): high but differing band powers between hippocampus and BLA are associated with lower TC levels (leftmost basin), whereas lower, but seemingly more synchronous band powers are associated with higher interaction values (rightmost basin). Stimuli encounters are noticeably absent from the third basin, which is homogeneous in neuronal activities and movement (“BLA rate,” “Hpc Rate,” and “Speed” annotations) but shows no sign of significant SWRs and TCs.

To aid comparisons across animals, we devised a matching procedure that pairs PI regions of Rat1 and Rat2 (Extended Data Figs. 4-2, 4-3, bottom bars) to the four basins of Rat3 (colors are matched). The procedure is based on similarities between the external annotations, e.g., “NREM” or “Hpc rate”, and relies on the clusters identified from the Sapphire plots (Materials and Methods, Matching of states across rats). These elements will be described in detail below. For Rat2 (Extended Data Fig. 4-3), the association provides a landscape similar to Rat3 for the red and green basins, with a mixing between the TC-active yellow and blue states in the intermediate PI regions. Conversely, the data from Rat1 (Extended Data Fig. 4-2) give rise to basins that correspond less to those of Rat2 or Rat3. Instead, despite the emergence of similar feature patterns visible in the TC and SWRs annotations, the algorithm appears to identify many smaller substates with sometimes very peculiar features (e.g., the tiny leftmost state). It is worth mentioning that Rat1 not only showed a reduced rate of crossings in the run epochs compared with Rat2/3 (∼0.3, ∼1.0, and ∼2.0, respectively) but also exhibited a ∼30-fold difference in this rate across sessions (∼2- to 3-fold for Rat2/3). This can be recognized clearly in the Sapphire plot (Extended Data Fig. 4-2) from the lower density of air puffs and reward points in the Time annotation. The Sapphire plot for Rat1 (Extended Data Fig. 4-2) reflects this variability in behavior. We stress that the matching algorithm is simplistic, in particular because of imposing a size constraint derived from Rat3 (see Materials and Methods, Matching of states across rats). We also emphasize that the order of states in a Sapphire plot is arbitrary. The role of the four-color highlighting is primarily to locate corresponding regions across animals quickly. Generally speaking, and this holds for all three animals, the time annotation shows that the individual experimental sessions all contribute roughly equally to a given coarse state although the recordings are almost certainly non-stationary across days because of the progressive lowering of the electrodes (see Materials and Methods, Experimental design and behavior). Importantly, within all of these aforementioned coarse states, the kinetic annotation suggests potential substates, and this is what we investigate next.

#### Clustering by the Sapphire plot and characterization of the identified states.

The Sapphire plot provides a comprehensive visualization of putative states along with the labels used to characterize individual points in time. Nonetheless, given the size of the dataset and the amount of labels to be considered at the same time, we deemed it desirable to compress the amount of information displayed. Toward this goal, we take advantage of the kinetic and time annotations to formally identify clusters consisting of time points that are both kinetically and structurally close. The method is called SbC given that clusters originate directly from the partitioning of the PI sequence into chunks (see Cocina et al., 2020; Materials and Methods, Clustering from the Sapphire plot, as well as Extended Data Figs. 4-1, 4-4). Specifically, we combine the PI ordering with the temporal information as found in both the Kinetic and Time annotations. In the former, the partitions are identified as peaks in the Kinetic curve, whereas in the latter “blocks” of distinct temporal patterns are identified from an underlying 2D histogram (Extended Data Fig. 4-1*B*, panels 2, 3). Eventually, a consensus is reached across the results from the two annotations. The number of clusters cannot be set directly but is controllable monotonically through the size of the bin in the aforementioned histogram, which sets an upper limit for the resolution of small clusters (see Materials and Methods, Clustering from the Sapphire plot and Cocina et al. (2020) for more details). It is important to note that we exclude external annotations, e.g., the labels on the sleep phases, to arrive at the partitioning. Given that the original dataset of TCs and band powers is what defines the PI ordering, we can characterize each extracted cluster by a particular combination of internal state values, i.e., the LFP power bands and reciprocal interaction patterns between hippocampus and BLA (Fig. 5*A*). Of course, the definition of a single fingerprint per cluster masks the heterogeneity within it, which means that a relatively fine partitioning is required for these fingerprints to be informative.

Once the clusters have been identified, we assign, to each of them, an affinity score related to the labels that annotate the Sapphire plot (Fig. 4). These results are plotted with the help of the unfolding method, a dimensionality reduction technique that can project both clusters and labels onto a 2D space (Cox and Cox, 2000; Borg et al., 2018). This procedure, in good part inspired by previous traditional MDS applications (Shinomoto et al., 2009; Kyriazi et al., 2018), provides an effective visualization of the distribution of the states across the different labels. The unfolding method is an MDS technique in that it aims to minimize a stress function that accounts for the differences between the distance matrix in the original space and the one in the reduced space (see Materials and Methods, Unfolding projection). It is worth clarifying how the distance matrices differ between the unfolding technique and traditional MDS. In the latter, one would compute, e.g., Euclidean, distances between *n* clusters using affinity scores for individual labels as coordinates in an *m*-dimensional space, thus projecting only the clusters onto the reduced space. In the unfolding case, the *n* clusters and *m* labels are both considered as points to be projected. A distance matrix is assembled that relies only on affinity scores to control the placement of clusters relative to labels but does not supply data to the stress function that contains information about distances among clusters or among labels.

Unfolding projections for the three rats are shown in Figure 5*B–D*. Across all three animals, the first thing to note is that there are two (nearly) consistent clusters of labels: Speed, Run, and Wake versus NREM, Sleep, Post-Sleep, and SWRs. The remaining four labels (Hpc Rate, BLA rate, REM, and PreSleep) are more volatile. To understand the placement of clusters relative to labels, it is important to realize that the labels are not mutually exclusive, e.g., NREM must coincide with Sleep and spans both PreSleep and PostSleep labels. Thus, it is impossible for a cluster to coincide exactly with a single label. This limitation leads to the observed distribution, which is fairly uniform across the plot. However, we can assert that for at least two rats the placement does show an affinity with specific labels, which is highlighted by the coloring according to PI position as seen, in particular for Rat2 and Rat3, by comparing the Sapphire plots of Extended Data Figure 4-3 and Figure 4 with the unfolding projections of Figure 5*C*,*D*, respectively. For these two cases, the clusters from the left of the Sapphire plots (Fig. 5*C*,*D*, light colors) are clearly associated with NREM phases as expected from Extended Data Figure 4-3 and Figure 4. In addition, for Rat2 there is an accumulation of clusters shown in gray near the REM label (Fig. 5*C*), which is consistent with the higher prevalence of REM time points in the intermediate regions of the PI of Extended Data Figure 4-3. In all three panels, the layout suggests that the first dimension (horizontal) resolves a transition between awake/active and deep-sleep (NREM) phases. Since such a distinction can be inferred by the power band values alone, we conjectured that the second dimension might be related to the overall level of TCs. In order to assess this in a collective picture for all the rats, we performed the unfolding on the dataset combining all three affinity matrices. A so-called biplot is shown in Figure 5*E* where the direction of enhancement of TC is indicated by arrows (see Materials and Methods, Unfolding projection). Each arrow indicates the average influence of individual bands, pointing toward the direction of larger enhancement across the low-dimensional space. The TCs that are significantly mapped in reduced space point downwards and overlap, especially for most of the hippocampus → BLA interactions. This confirms that “MDS-2” (i.e., the vertical dimension) is predominantly an overall interaction axis (high at the bottom, low at the top). In conjunction with the placement of labels, this is consistent with the results presented above (see Interaction analysis between behavioral epochs and sleep phases).

#### When quantifying the ability to separate labels, the SbC method shows the highest fidelity among four tested methods and TCs effectively contribute to a better classification.

It is useful to question whether the analysis in Figures 4, 5 yields putative clusters that do in fact describe the internal state of the joint hippocampus-BLA system. Unfortunately, in the absence of a ground truth, the only feasible way to gather evidence for this is to quantify how well states tend to separate labels, e.g., behavioral annotations. Here, our proxy for performance are the stress values from the unfolding technique, which measure the degree of both the quality of the projection and the ability of the state partitioning to sort out the different labels (see Material and Methods, Affinity scores). Extended Data Figure 6-1 shows that the volatility of certain label positions in Figure 5*B–D*, in particular BLA rate and PreSleep, is mirrored in their high relative stress contributions for Rat1 and Rat2. Generally speaking, most labels appear to make similar contributions to the stress, however.

Such a global measure of performance also allows us to compare the clustering obtained by SbC to those obtained with other methods. In Figure 6*A*, results from this comparison are shown for three clustering techniques commonly used for large datasets. In all cases, the number of clusters was set equal to that obtained with the SbC method since stress values tend to depend on the number of points to be projected. As an additional test, we were also interested in the effective contribution of the TCs in separating the labels. To do so, we performed the SbC algorithm on a dataset not containing TCs. The data show that the SbC achieves lower (i.e., better) stress values with respect to the other clustering methods. We also note that neglecting TCs results in poorer performance in two rats and in comparable values for the remaining animal, indicating that TCs are helpful in deciphering the coding in these brain areas. It is a caveat that the number of clusters differed slightly (it is not a direct parameter in the SbC algorithm, see Materials and Methods, Clustering from the Sapphire plot). The relevance of TCs collected as a function of time was examined in two random permutation tests (Fig. 6*B*). In the first, the time bins of TCs were shuffled in blocks, which maintains their local patterns but mismatches them with the band powers’ time points. In the second, we permuted the TC identity at each time bin instead, which mismatches the various pairs of frequency ranges with respect to the band powers. In two rats and for both distributions, the true values indicate a significant role of TC coordination in discriminating the labels. For the remaining rat, coherently with Figure 6*A*, TC does not seem to provide improved performance. However, overall, we conjecture that the main contribution is provided by the LFP powers, which is reasonable given that many labels are directly correlated to it, e.g., NREM or REM phases or the presence of SWRs.

10.1523/ENEURO.0484-20.2021.f6-1 Extended Data Figure 6-1 Analysis of stress values of the unfolding projection. Top, middle, and bottom, Results for Rat1, Rat2, and Rat3, respectively. Percentage of total stress values per unfolded label. Download Figure 6-1, EPS file.

10.1523/ENEURO.0484-20.2021.f6-2 Extended Data Figure 6-2 Results of the unfolding projection applied to the combined dataset with alternative preprocessing. The Sapphire plots from which the clusters are derived are shown in Extended Data Figures 4-5, 4-6, 4-7. A–C, Unfolding plots for Rat1, Rat2, and Rat3, respectively (analogous to Fig. 5B–D in the main text). D, Unfolding projection for all three rats and biplot of the relevant mean TC components (analogous to Fig. 5E in the main text). E–G, Stress per label, comparison with other clustering methods, and robustness with respect to TC shuffling (analogous to Fig. 5F, Extended Data Fig. 6-1A,B). Download Figure 6-2, EPS file.

#### Robustness of the Sapphire methodology against alternative preprocessing

We tested the proposed analysis, which combines the Sapphire plot with an unfolding method, also on an additional featurization with fewer dimensions (5) and alternative preprocessing (see Materials and Methods, Combined dataset of LFPs and TCs). Extended Data Figures 4-5, 4-6, 4-7 suggest that in this case the Sapphire plots for the three rats are dominated by the band powers, which gives rise to a better separation of sleep phases and the appearance of a more detailed kinetic annotation. The strong clustering of “reward” labels in Extended Data Figure 4-7, which is not based on simple features like net BLA activity, is a striking example demonstrating that changes to the pipeline will be able to uncover further insights from these data in future work. There are two main reasons why we prefer the pipeline used for Figure 4 here. First, the original dimensionality (18) is such that TCs play a significant role in identifying clusters. Conversely, in Extended Data Figures 4-5, 4-6, 4-7, TCs appear to not be strongly represented in the retained five principal components. Second, many of the smaller clusters suggested by the kinetic annotation have no apparent interpretation because of the coarseness of the available labels, which makes their interpretation challenging. From Extended Data Figure 6-2, it is clear that both preprocessing pipelines give similar ranges of stress scores, thus offering no reason to favor either. However, it is interesting to note that the results for the contribution of TC to the stress values are inverted across rats. Whereas the data for Rat2 fail to establish TC as substantial for reducing stress in Figure 6*B*, exactly the opposite is the case in Extended Data Figure 6-2*F*, where only Rat2 establishes TC as significant. Because we could not find hints in either behavior or TC values that could explain this dissimilarity with the other rats, we conjecture that the individuality of animals might require preprocessing pipelines to be tailored toward each animal, at least when the goal is to maximize sensitivity. Overall, however, two rather different pipelines (unweighted vs dynamically weighted features, dimensionality of 18 vs 5), produce Sapphire plots that preserve, at a coarse level, both the mutual similarity of coarse states (like NREM sleep) and, approximately, the stress levels in the embedding. Thus, these data highlight the robustness of SbC, in particular relative to the three approaches used for comparison.

## Discussion

We propose here a set of methods for an unsupervised investigation of neural states and interaction patterns in complex datasets featuring recordings from multiple brain regions. We employed these methods to conduct a global and exploratory analysis of directed interactions between hippocampus and BLA, which relies on signal power alone. From the recorded LFPs, we extracted band powers in six different frequency bands and analyzed them in a three-stage approach. The dataset allowed us to conduct an extensive investigation of reciprocal influences between the two regions and to identify underlying neural states characterized by different levels and patterns of interactions across multiple epochs (Girardeau et al., 2017b). The methodological pipeline we presented above comprises unsupervised and scalable methods that take advantage of the entire recorded time series (see Materials and Methods, Progress index (PI) and Sapphire plot). As such, our methodology is well-suited to a wide variety of complex neural data when the primary goal is an exploratory analysis of recurrent neural patterns. The three stages were: (1) a canonical comparison of interactions between regions across different behavioral and physiological time periods; (2) a high-resolution visualization procedure adopted from methods for the analysis of molecular dynamics simulations and (3) a global inference of putative neural states from a dataset combining time-resolved measures of reciprocal communication between the two brain regions with region-specific band powers. In the following discussion, we first examine benefits and limitations of the tools and workflow used during the analysis. We then focus on the biological insights that our analysis offers by recapitulating its salient aspects as well as discussing similarities and discrepancies with previous literature results.

### Methodological and theoretical insights

#### Directed interactions and the use of TC

We chose TC (Harnack et al., 2017; Fig. 1*C*,*D*; Materials and Methods, Topological causality) to capture, at each time frame, potential bidirectional interactions and to quantify asymmetries in the strength of these reciprocal influences. In the presence of cycles or clear nonlinearities in the flow of information, it is probably more appropriate to analyze the system as a whole, that is, to view the time evolution of a variable *Y* not merely as the sum of its present state and external inputs from *X* but rather as the result of the joint history of the values of both *Y* and *X*. This is related to the concept of non-separability of the system, which signifies that the history of *Y* contains redundant information about *X* that cannot be isolated and formally removed from the equations of motions of *Y* (Sugihara et al., 2012). Thus, it is better to adopt here the notion of interdependence or “generalized synchrony/synchronization.” This concept was established in multiple works in which the authors developed approaches to robustly quantify interdependence based on expansive mapping (Arnhold et al., 1999; Quiroga et al., 2000; for a review, see Pereda et al., 2005). These methods and TC have been applied to EEG data (see also Stam, 2005; Tajima et al., 2015, 2017), and their results encouraged the present application of TC to LFP recordings in the hippocampus-BLA system.

Many sophisticated methods provide valuable alternatives to evaluate nonlinear patterns of influence, such as nonlinear GC, dynamic causal modeling, and transfer entropy (Schreiber, 2000; Friston et al., 2003; Chen et al., 2004; Ishiguro et al., 2008), which, importantly, rely on different paradigms of functional connectivity estimation (Friston et al., 2013). However, we work with an actual biological dataset, and the absence of a ground truth makes it difficult to offer a sufficiently informative comparison across methods. Some comparative evaluations performed on test datasets can be found in the literature (see Gourévitch et al., 2006; Harnack et al., 2017; Krakovská et al., 2018).

The data-inferred interdependence and reciprocal influence between the two components of a nonlinear system differs from the event-driven cause and effect assessment performed by neural intervention and manipulation (Harnack et al., 2017; Jazayeri and Afraz, 2017). In the latter, a stimulus defined by the experimenter serves a causative role for (temporally) subsequent observations (the effect). This type of causation is typically deemed unequivocal as long as proper controls are in place. The main limitation is that most stimuli or interventions will only relate indirectly to the function of the brain. In contrast, a data-driven inference of directed interactions can in principle address and study functional units of the brain directly. In fact, these interactions could even be estimated accurately in a cause and effect sense if we were confident that our time series contain all the information necessary to fully describe the system’s dynamics (Granger, 1980). However, this is an unrealistic scenario both in general and for our analysis here given that we are able to observe only two brain regions at a time. If our data are not complete in the above sense, results have to be interpreted with caution. For example, some of the identified interactions may simply be the result of an external signal “passing through” in sequence. In other words, a driven interaction in the BLA-hippocampus system need not indicate an important role for either region in the process being observed. That said, TC does go beyond linear models of connectivity like GC and CCF, as, unlike those, it accounts for the non-separability of the system and can help elucidate details of the information exchange between hippocampus and amygdala (Sugihara et al., 2012; Harnack et al., 2017). The fact that some but not all of our results are congruent with prior results (Popa et al., 2010; Stujenske et al., 2014; Girardeau et al., 2017a), as detailed below, lends credibility to this mesoscopic, interaction-centric paradigm of analysis.

#### On the use of spectrograms

The use of multitaper spectrograms, the extraction of band powers from these, and the application of a quantile (rank) transformation on the time-resolved power values allowed us to compute TCs with globally consistent settings (see Materials and Methods, Topological causality). However, the adoption of this widespread filtering operation carries obvious downsides, in particular related to a potential loss of information and the blurring of interactions (de Cheveigné and Nelken, 2019). Specifically, as spectrograms average across a time window, here 2 s, information on temporal details is lost, such as phases, or can be smoothed out in time, such as variations in amplitudes and details of SWRs. This blurring adds uncertainty to the localization in time of the interaction, and, generally speaking, can alter the apparent temporal precedence between two time series. However, as explained in Materials and Methods, Alignment settings, we tried to minimize the likelihood of such artifacts by deriving interactions from time series that we obtained with the same time-frequency analysis: the same spectrogram window was used for all the bands, and we maintained a consistent alignment between the resulting band powers throughout for deriving TCs (de Cheveigné and Nelken, 2019).

#### On studying only power-power interactions

The study of cross-frequency and high-frequency interactions largely involves phase-related measures, such as phase-amplitude and phase-phase couplings, as well as coherence (Lisman and Jensen, 2013; Fries, 2015). Whereas these analyses investigate fast timescales (∼10 ms), which are commonly interpreted to report on genuine physiological mechanisms, power-power or amplitude-amplitude interactions, which we study here, have been examined less often as their functional significance and mechanistic modalities are less clear (Canolty and Knight, 2010; Siegel et al., 2012). The higher timescales associated with these types of interactions (∼0.1–1 s) were observed in cross-frequency co-modulations between θ and γ band powers in the hippocampus in Shirvalkar et al. (2010) and Buzsáki et al. (2003) where the authors employed similar spectrogram windows as those used here. In addition, it has been reported that trains of contiguous SWRs are detectable at these higher timescales (Fernández-Ruiz et al., 2019). Thus, the construction of spectrograms with its accompanying change in information content, helped by the nonlinear nature of TC, allows the inference that the interactions reported in this work might differ from and complement the ones found through a phase-based analysis. Clearly, our analysis is best-suited to identify mesoscopic interactions that are related to the generation of sustained rhythms, of sequences of burst events and, generally, of large irregular activity (Davidson et al., 2009; Palmigiano et al., 2017; van Ede et al., 2018; Fernández-Ruiz et al., 2019; Zich et al., 2020). This does not allow the inverse conclusion that other phenomena cannot be observed at all, however. As Figure 1*E* shows, the resultant TCs do in fact vary quickly in time, which is further enhanced by the fact that we zero non-significant values in the TC time series (see Materials and Methods, Topological causality). In summary, our analysis goes beyond the (large) part of the literature that deals with (filtered) signals at higher resolution and focuses on coherent oscillations.

#### Advantages of the proposed methodology for the identification of neural states

We combined the information provided by the band powers and TCs to search for a possible temporal recurrence of interaction patterns. To this end, we concatenated data from multiple recording sessions for each of the rats, thus considering a large dataset (∼10^6^ time frames). Importantly, we did not apply any pre-selection of temporal windows to be analyzed by, e.g., restricting ourselves to sleep phases, SWRs frames, or periods with high firing rates. Moreover, the assessment of clustering performance employed a set of available labels commonly adopted to characterize individual frames in these time series. The SbC method performs better than three commonly employed clustering tools for all the animals analyzed, and this remains valid even if the data are preprocessed differently (Extended Data Fig. 6-2). We hypothesize that the main advantage with respect to other techniques consists in using explicitly the temporal information. This is done by evaluating both the recurrence of the states along the time series and the actual kinetic distance between them. Importantly, we showed that TC provides in most of the cases a significant contribution in discriminating the different behavioral and neural labels that annotate the recordings. Generally, there are no particular limitations in extending application of the Sapphire plots and subsequent analyses to other types of neural dataset with different experimental settings (see also Garolini et al., 2019). For example, in a typical working memory experiment, the animal repeatedly performs a task with fixed temporal structure under different contexts. The Sapphire plot resolves this single-trial structure and may thus be used to investigate whether any of the trial epochs allow a better distinction of one or more of the different contexts.

### Biological insights

#### Physiological connection of hippocampus and BLA and biological limitations of the analysis

The ventral part of the hippocampus projects directly to the BLA, in contrast to the dorsal one, which is what is studied in the original dataset and analyzed here. However, as remarked in Girardeau et al. (2017a), the interactions between the dorsal hippocampus and the BLA could be mediated by indirect pathways, with particular attention given to the entorhinal cortex (Bauer et al., 2007; Colgin, 2016). In this regard, it is also important to note that our analysis relies only on the LFP signals, across all bands, and does not use spike information. Coordination between LFPs in the dorsal hippocampus and the BLA has in fact been observed in the θ (Seidenbecher et al., 2003; Bienvenu et al., 2012) and high-γ (Stujenske et al., 2014) bands, and the results of Girardeau and colleagues highlight the importance of SWRs for such coordinated activity. Importantly, the nature of the data and the interactions we are able to analyze address purely the issue of functional connectivity between the two regions rather than the issue of direct projections.

There are further considerations that might limit the biological interpretation of the results obtained as follows. In our analysis, band powers or other features of the LFP signal have not been thresholded to single out selected oscillatory events. Given this, the known 1/f trend in the LFP spectrum in a broad and heterogenous state such as NREM should be accounted for. Similarly, it has been observed that β and part of the low-γ oscillations in the hippocampus arise from overlapping effects of SWRs during NREM sleep (Oliva et al., 2018). Finally, volume conduction might interfere with signals in low-frequency bands (Łe ¸ski et al., 2013). Specifically for the δ and θ bands, we tried to estimate its role in the Results section (see Interaction analysis between behavioral epochs and sleep phases) and in Extended Data Figure 2-2: while we can reasonably deduce that the contribution is marginal for θ, we did not arrive at the same conclusion for δ. In particular, oscillations traveling from the hippocampus to the BLA via volume conduction (Bertone-Cueto et al., 2020) pose the scenario that BLA δ-driven interactions detected via TC are actually indicative of activity and interactions within the hippocampus alone. All of these qualifying considerations should be kept in mind when interpreting some of the details of our results.

#### Interaction patterns in REM and NREM sleep

One of our main observations is that reciprocal influences between hippocampus and BLA are particularly sustained during NREM sleep and that this mutual influence is carried predominantly by the low-frequency bands, namely δ, θ, and β. During REM sleep, a general reduction of interactions is observed in these bands and interareal communication appears to be mediated by the respective fast frequencies. The notable exception to this are hippocampus-driven TCs in the θ band, which are actually stronger than in NREM sleep. We conjecture that the more complex interplay of different frequencies, along with the shorter duration and lower regularity of activity in REM sleep, is the likely culprit for the apparent lack of correlation between the various interaction measures in Figure 2*B*,*C*, bottom panels. While the slightly larger differences between pre-sleep and post-sleep appearing in NREM with respect to REM sleep (Figs. 2*A*, 3) appear to corroborate the findings of the original analysis of the same data (Girardeau et al., 2017b), our results for REM sleep are at odds with the role REM sleep is commonly assigned to play in consolidating emotional memories (Genzel et al., 2015; Hutchison and Rathore, 2015; Boyce et al., 2016). For example, the work of Popa et al. (2010) demonstrated that an increase in the θ coherence during REM between hippocampus, BLA, and medial prefrontal cortex (mPFC) correlated with the consolidation of conditioned fear. In agreement with their Granger analysis, we do observe an apparent, mostly one-sided influence of the hippocampal θ on the BLA θ band (Fig. 2*A*). If we extend the view to other frequency bands, we find that the BLA θ influence on various hippocampal bands decreases significantly with respect to NREM sleep whereas the hippocampal θ band is significantly enhanced also in its influence on higher BLA frequencies. However, and this differs from expectation, we do not observe any significant contribution of the θ band, in either region, to mark differences between pre-sleep and post-sleep REM epochs (Fig. 2*A*). This is despite the fact that the BLA θ-driven TCs are one of the channels that appears to be able to distinguish pre-run from post-run in the aversive direction (Extended Data Fig. 2-1).

In another work (Stujenske et al., 2014), high-γ BLA activity during wakefulness was observed to drive ventral hippocampal high frequencies during safety signals. In contrast, we do not observe a specific pattern for the safe direction (Extended Data Fig. 2-1), which is probably expected given the established differences in the roles of the ventral and dorsal regions of the hippocampus (Fanselow and Dong, 2010). During NREM sleep, reciprocal interactions between the two lowest frequency ranges may originate instead from the sequential processing in hippocampus and BLA of slow oscillatory signals originating from the cortex (Genzel et al., 2014, 2015). Interestingly, many of the significant differences between post-sleep and pre-sleep episodes or between aversive and safe directions concern communication from the BLA to the hippocampus with the β band of the amygdala playing a prominent role (Fig. 3; Extended Data Fig. 3-2). To our knowledge, β oscillation bursts in the amygdala have been characterized in detail only in Stujenske et al. (2014) where they were triggered by an auditory cue preceding an aversive stimulus. Differently from that work, the data we analyzed are from experiments where rats perform a non-Pavlovian spatial task. It is thus difficult to provide an exact side-by-side comparison of our results for the β band to those of Stujenske et al. (2014).

#### The role of SWRs

We provide additional evidence that SWRs are a hallmark of episodes in which the hippocampus exerts a directed influence on the BLA whether during sleep or not (Fig. 3; Extended Data Fig. 3-2). Interestingly, the TC analysis indicates that this influence is not confined to the ripples band but also mediated through the δ, θ, and β bands. SWRs play a prominent role in memory consolidation and retrieval, since they seem to provide a mechanism for facilitating the transfer of hippocampal memories to connected brain regions (Girardeau and Zugaro, 2011; Buzsáki, 2015; Joo and Frank, 2018; Laventure and Benchenane, 2020). As shown in Girardeau and colleagues, hippocampal SWRs upmodulate precisely those BLA cells that contribute the most to the joint neural reactivations in post-NREM sleep. In our analysis, while SWRs are a significant indicator of directed interactions with the BLA driven by the hippocampus (Fig. 3), there are barely any significant differences in the interaction patterns, neither when considering the entire pre/post-sleep epochs (Fig. 2*A*) nor when restricting the analysis to NREM sleep (Fig. 3). To us, the most likely scenario explaining this lack of contrast is threefold. First, the experiment employs ubiquitous positive stimuli in the form of water rewards. They are present whenever the rat is on the track (Fig. 1*A*) and might provide a strong background signal of reciprocal communication. Second, the only clear evidence we see of contrast between pre/post-epochs is for the pre/post-run phases (Extended Data Fig. 2-1), which differ only in that the negative stimuli were encountered more recently (time scale of 2–3 h) in the post-run phase. This suggests that the timing of memory retrieval/processing is specific. Third, our power band-based analysis might lack the necessary sensitivity, in particular in the ripples band.

#### A functional view of neural activity in the form of well-defined states

The time series analyzed, i.e., LFP power band contributions and the TCs derived from them, belong to a mesoscopic level of observation. This representation of the neuronal state of the joint BLA-hippocampus system provided us with a coarse characterization of the system. For example, in Figure 4 (Rat3), we identify, in an unsupervised manner, four coarse states (from left to right): NREM sleep with low interactions but high SWR activity; activity involving emotional processing (both reward and fear) with directed interactions but low SWRs; a waking but seemingly inactive state with neither interactions nor SWRs; finally, a mixed state of NREM sleep and activity with emotional processing where both the reciprocal influence and SWRs are high. The (few) REM time points are embedded in the first active state (second from the left), and, to a lesser extent, also in the last mixed state, which is consistent with the well-known similarities between neural activities in REM and wake phases. The last (rightmost) state is the most interesting one given that it features mostly SWRs, NREM sleep, and both aversive and reward events while carrying a signature of strong interactions. This latter state is a potential candidate for addressing the joint neuronal reactivations that capture the emotional and contextual components of the run epoch (Girardeau et al., 2017a). However, since we could not find a clear distinction between pre-sleep and post-sleep epochs in our unsupervised analysis, the data suggest either that the discriminating patterns are too sporadic to create well-defined states, or that information about spiking activity is of critical importance and must be accounted for in the dataset.

In the spirit of an exploratory analysis, we did not formulate a specific hypothesis in this work. We rather adopted a set of computational tools, some of which were originally developed for completely different tasks, for an unbiased, data-driven exploration of mutual interactions between amygdala and hippocampus across different types of neural activities. Because we use band powers rather than raw LFPs to infer directed interactions between these two brain regions, our analysis is focused on function at mesoscopic resolution rather than on anatomic connections at the level of specific groups of neurons. The ultimate scope of the results presented here is to suggest new hypotheses to be tested by confirmatory research (Schwab and Held, 2020). For example, we hypothesize a role for the BLA β band in promoting memory consolidation (Fig. 3), which is putatively associated with the processing of negative stimuli (Extended Data Fig. 3-2). Similarly, based on the third coarse state in Figure 4, we conjectured that there is a neurologically homogeneous “resting” state in which amygdala and hippocampus are, except for a striking absence of SWRs, active but decoupled. We hope that our work will help motivate future analyses and experiments aimed at testing hypotheses like the above.

## Synthesis

Reviewing Editor: Mark Laubach, American University Decisions are customarily a result of the Reviewing Editor and the peer reviewers coming together and discussing their recommendations until a consensus is reached. When revisions are invited, a fact-based synthesis statement explaining their decision and outlining what is needed to prepare a revision will be listed below. The following reviewer(s) agreed to reveal their identity: Éléonore Duvelle.

Your manuscript was reviewed by the two original reviewers. One felt that your changes were satisfactory, but still had some concerns about the manuscript. The other reviewer was not satisfied with the changes that were made. Specifically, they were concerned that your method “should be compared with existing approaches to better understand its inferential power or limitations", and felt that your revisions did not sufficiently address this issue.

We decided to bring in a third reviewer. An expert in the focus of your work (brain regions and methods) agreed to review the paper, and their review is provided below in full. Please address all points that were raised in their review in your revised manuscript. The third reviewer and I will review your revised manuscript.

The consensus from our discussions of your manuscript is that you should focus on highlighting the method over the results in the revised text. Your method was judged to be novel but the experimental results were not viewed as a major advance to the field.

Reviewer #3

Advances the Field: This manuscript has the potential to advance the methods used in the field, but in its current state, does not seem to provide clear advances in terms of knowledge about the brain.

Statistics: From the explanations provided in the manuscript, it was unclear to me how the main analysis used by the authors - ‘topological causality’ - works. Better control situations could probably be used but it is difficult to suggest any without knowing what the analysis does exactly. The authors claim that they are testing causality in some places and mention that they can’t really assess causality in others. However these concerns might disappear with better explanations of the method and its potential limitations.

Summary:

The authors reanalyse an existing dataset containing extracellular freely-moving recordings in the dorsal CA1 hippocampus and basolateral amygdala of rats (Girardeau et al., 2017). The recordings were done during different sleep stages (REM and non-REM) and also while the rats were running between two ends of a linear track; in specific trials, one direction of the run would trigger an air-puff, aversive for the animal. This dataset is well-suited to the study of cross-area communication related to the learning and consolidation of aversive experiences. The authors note that the present study is exploratory and they do not have any a priori hypotheses.

The authors employ a two-step analysis. First, they use a method that seems relatively novel in the neuroscience field, called ‘topological causality’ to evaluate the putative influence of one brain region over the other. In this case the method is applied to the power of specific oscillations in the local field potential (delta, theta, gamma, ripple etc.). The authors find specific patterns of interactions between the hippocampus and the amygdala (usually termed ‘causal’ in the manuscript) during specific stages of the experiment. The method is compared to previously used ones such as Granger causality and is found to have similar or different outcomes depending on the sleep stage.

In a second part, the authors employ another potentially new method to visualise and classify activity patterns reordered in an unsupervised manner, using a measure called the ‘Progress Index’. This method uses as an input the principal components of a data set comprising of 1) the LFP power at the different power bands stated above and 2) the different types of topological causality computed from the LFP. The reordrered data obtained is then formally classified using ‘Sapphire-based clustering’ and the performance of the classification method in finding behaviourally-relevant states is evaluated by comparing the clusters to ground-truth labels of the data (when available). The new method is shown to be better than other clustering methods and than chance at finding such clusters for 2 out of 3 rats.

General Strengths & Weaknesses

The authors’ effort to apply these complex methods to a relatively complex data set is impressive and should be applauded. These methods might be of interest to the neuroscientific community which has a growing need for tools allowing unsupervised analysis of large and multi-area datasets. Despite the complexity of these analyses, the process remains relatively understandable, thanks to the didactic figures and explanations provided in the manuscript; however, this could still be improved further. I also appreciated just looking at the Sapphire plots, even though it is difficult to extract any strong conclusion from them. Thus, this manuscript provides a potentially interesting methodological contribution to the field.

On the other hand, the contributions of the manuscript in terms of neuroscientific knowledge (especially beyond that of the original paper, Girardeau et al., 2017) are unclear to me as it does not seem to provide any significantly novel insights about the brain, and it does not test a specific hypothesis (acknowledged by the authors). The authors do propose a new hypothesis at the very end of the discussion (role of the basolateral amygdala beta band in memory consolidation) but, from the wealth of data analysed, one could have expected a lot more. It also has some drawbacks, namely, 1) it is unclear how the ‘topological causality’ analysis can provide any information about causality, as no causal manipulation is done in the experiment; 2) most of the results of the unsupervised classification of states of interest can be quite simply explained by the difference of power in theta and ripple band in the different states (high theta in REM/running, low theta in nREM sleep or rest); 3) the results are quite variable across subjects (usually replicating for only 2 of the 3 rats) ; 4) the analysis might not be suited for very fast oscillations such as hippocampal ripples; 5) volume conduction could perhaps explain some of the results. However, points 2 to 4 are already discussed by the authors and the other points might be relatively easily clarified.

In summary, I believe this manuscript is likely to be of interest to the readership of eNeuro looking towards using new analysis methods provided the main concerns are addressed by the authors - detailed below.

Major concerns

1. ‘Topological causality’ analysis:

a. While there are quite a few panels attempting to explain how this analysis operates (which do help), the paper would greatly benefit from a more intuitive explanation of this measure in simple terms so that even a naive reader can evaluate its validity. What exactly is it measuring? What does the term ‘topological’ stand for in this context?

b. Related to this, it is unclear how this analysis manages to extract ’causality’ between two brain regions from, in this case, the power of LFP oscillations, since there is no manipulation of neural activity. The only explanation I can think of is that the analysis measures how well activity in region X predicts activity in region Y, with some delay supposedly reflecting transfer of neural activity. This is not proving causality as both regions could act this way independently, either by chance, because they are driven by similar sensory stimuli, or because they receive inputs from the same brain region. If this is all that the analysis does, I would suggest that the authors remove the mentions of ‘causality’, ‘driven by’, ‘causal links’, ‘causal patterns’ etc. throughout the manuscript, while keeping the term ‘topological causality’ as it is the name of the analysis. The authors already discuss this and acknowledge the fact that they cannot be certain about any detected causality, but there are still instances in the manuscript that use the term. Ideally, a control with an obvious absence of causality would be useful, for example using the hippocampal data from one animal and the amygdala data from another, to assess what would be the baseline level of ‘causality’ found by the analysis. However, at this stage, it seems simpler to avoid implying findings of causality.

2. The possible effects of volume conduction of the different LFP rhythms should be discussed (e.g. Bertone-Cueto et al., 2020, https://www.frontiersin.org/articles/10.3389/fnsys.2019.00078/full “Regions in the convex side, such as visual, auditory and entorhinal cortices, amygdala, colliculus, and even some mesopontine regions would also receive volume-conducted hippocampal theta of smaller amplitude.” ). Could oscillations generated in the hippocampus also be detected in the amygdala? How does the analysis prevent interpreting this as a ‘causal’ influence of hippocampus on the amygdala? Fast oscillations like ripples are likely to propagate less, but because the analysis is less appropriate for these oscillations (which are much faster and transient than the timescale used in the analysis), the most interesting findings will be for slower oscillations and these seem more likely to spread through brain tissue.

3. Is the topological causality measure more likely to find ’increased causality’ in a given band where the power is higher (for example, increased theta during REM sleep compared to nREM sleep)? If yes, how do the authors control for this?

Minor concerns

1. The title and goal of the manuscript could be clarified to ensure that the reader’s expectations are met, perhaps putting more emphasis on the methodological aspect (something like: ’Unsupervised methods for detection of neural states: case study of hippocampal-amygdala interactions’), or, if the emphasis is put on experimental findings, state these.

2. While reading the paper it seemed obvious that any classification method using LFP as an input would be able to separate the different states (REM, non-REM, running, eating, etc.) simply from the different patterns of LFP in these different states (especially given that REM and non-REM are labelled using the delta/theta ratio). However, an interesting analysis is shown in Fig6 where the contribution of the TC values is assessed and found to significantly improve the classification (for 2 of the 3 rats). This shows that there is information in the interactions between the two brain regions beyond their independent activity. I feel that this could be emphasized in the introduction instead of having to wait until the last figure to discover it.

3. When reading the paper in order (methods first), many concepts are not explained (e.g. what data is actually analysed, what is considered a state, what is a cluster, what is Taken’s theorem, what is time-lagged embedding, what does ’topological causality’ mean, which manifold is referred to L106? etc.). These are mostly clarified once the reader reaches the relevant part in the results, still, an introduction of the different concepts mentioned in the methods when they are first mentioned would be welcome. Several of my other comments below will probably be related to this.

4. Figure 1-1 (and also 4-1): links between what is shown in the figure and what is explained in the legend could be generally improved. If specific terms are used for the first (in the order of the manuscript) time in the legend, they should be explained. When specific signs, lines, colors etc. are used in the figure, they should be explained in the legend.

a. in Fig 1-1, where is m in the figure? What are the differences between time points in the embedding? What is the simplex projection method? Why do the time-series need to be aligned since they are already using a common time reference (t)? What do tau HPC(m-1) and tau BLA(m-1) mean, what do the two thin dotted lines represent?

b. In Figure 4-1: new terms should be explained. “peaks not satisfying specific selection criteria are rejected”: what are these criteria?; “partitions from (3) and (4) are eventually combined”: maybe the authors mean 2 and 3?

5. In figure 4-1’s legend, why is the significance threshold chosen to be p<0.01 while it is p<0.05 in all other analyses?

6. L75 “Only LFPs from the right basolateral amygdala (BLA) were used in the analysis”: why is this the case and how might this influence the analysis? Are the hippocampal signals bilateral? Does this change between rats?

7. L79 could the authors make it extra-clear which of the manuscripts’ rat names correspond to the names used in the original paper?

8. L86: ’the identification of different sleep phases (NREM/REM) was done with the help of a k-means clustering of the ratios of the delta to theta powers in the hippocampus’ Could the authors indicate which ratios (higher theta or higher delta) correspond to which sleep phase (NREM or REM)?

9. L87 why are the frequency limits for the same rhythm different for different brain regions (e.g. theta in hippocampus is 7-14Hz but 4-12Hz in BLA)? It looks like an explanation for this is given L311 (“in order to bridge distant frequency bands [...] we homogeneize the intrinsically heterogeneous timescales of variations in the lfps across the different band”), however, this explanation could be clarified.

10. L241 refers to two axes, but there is no figure referred to. Maybe the authors could refer to the example figure (4-1)?

11. L288 what does Mds mean?

12. L 322-323 “TC values that do not pass this significance threshold are zeroed explicitly (P-values <0.05)” perhaps the authors mean p-value>0.05?

13. In the example in Figure 1: “The actual values (solid lines) exceed the chance level [...] only for short, sporadic intervals” -> it would help to add stars or arrows to highlight these intervals.

14. L331 “low frequency bands”: please detail at least once, the first time they are mentioned, which of the bands are considered low-frequency. Also, it should probably be hyphenated (low-frequency bands’).

15. L377-378 “we partitioned the NREM data into patterns featuring SWRs and those that do not” It is menti0oned later that the SWR timestamps were provided in the original data set, but please explain how they were detected.

16. It would be informative to add a line for LFP in the theta band, or theta power, in figure 4 and if possible the other Sapphire plots (theta is likely to be a very strong predictor of the different states found).

17. L 434 “Comparing across animals, we can identify a similar set of basins for Rat2”: I do not find this very convincing - the plots seem quite different across animals. The only constant is that theta and ripple power influence the clustering. Maybe the authors could explain in more details the similarities across rats and in what way these similarities go beyond differences in LFP characteristics.

18. Figures with Sapphire plots for the different rats: it would help cross-rats comparisons if the axes shared a similar scale across rats (e.g. speed axis goes up to 60 for rat 3 but 30 for rat 2).

19. How is the starting point of the ’Sapphire plot’ determined? Isn’t the choice of start point likely to change a lot the states / basins found?

20. L451 How does SbC finds clusters and number of clusters?

21. Figure 5

a. legend: it is a bit unclear how the affinity scores are computed from that figure.

b. In plots b-d, how are the labels associated to some of the clusters? Why do only some clusters have labels?

22. L472 “it is clear that the placement does show an affinity with specific labels, which is highlighted by the coloring according to PI position as seen”: this is not very convincing (different colors seem to be all over the place), perhaps the authors could describe better what they mean.

23. The authors could discuss what makes rat 2 different from the others in terms of contribution of TCs to the clustering. Were the recording probes placed in different locations? Was the behaviour different?

24. L 590: ’tcs do go beyond linear models of causation like GC and CCF”: how?

25. L656 “they seem to provide a gating mechanism”: I’m not sure where this hypothesis comes from (it is not mentioned in the cited references). I suggest removing ’gating’.

26. Paragraph L669: It is unclear what is the point of this paragraph. The authors describe in qualitative terms the 4 states visually observed in one of the figures (for one rat) but do not attempt to explain what could be the functional relevance of these states; instead, they provide a relatively vague and rather obvious conclusion: “these causality patterns hint at memory consolidation mechanisms involving neuronal subpopulations.” Would this conclusion have changed had the authors found a different number of states or different types of states? What makes these states unique and interesting?

27. While the manuscript is generally very well written, there are a few typos eg. Legend of Figure 1-1 “The differences between time points in the embedding*are* dependent” ; L588 “have to interpreted” -> have to be interpreted.

## Author Response

We thank both the additional reviewer and the editor for their thorough evaluation of the manuscript and their valuable criticisms. As requested in the consensus/synthesis compiled by the editor, we have substantially increased the emphasis on the methodological aspect of the work. In particular, we changed title, abstract, and significance statement to clarify this. Throughout the text, additional information on the methods is provided, and a stronger emphasis is placed on limitations of biological interpretations.

Figure 2-2 has been added in relation to the reviewer’s comment on volume conduction. Simple deletions are marked with a strikethrough while modifications or additions relevant to the comments are highlighted in red.

------

Synthesis Statement for Author (Required):

Your manuscript was reviewed by the two original reviewers. One felt that your changes were satisfactory, but still had some concerns about the manuscript. The other reviewer was not satisfied with the changes that were made. Specifically, they were concerned that your method “should be compared with existing approaches to better understand its inferential power or limitations", and felt that your revisions did not sufficiently address this issue.

We decided to bring in a third reviewer. An expert in the focus of your work (brain regions and methods) agreed to review the paper, and their review is provided below in full. Please address all points that were raised in their review in your revised manuscript. The third reviewer and I will review your revised manuscript.

The consensus from our discussions of your manuscript is that you should focus on highlighting the method over the results in the revised text. Your method was judged to be novel but the experimental results were not viewed as a major advance to the field.

Reviewer #3

Advances the Field: This manuscript has the potential to advance the methods used in the field, but in its current state, does not seem to provide clear advances in terms of knowledge about the brain.

Statistics: From the explanations provided in the manuscript, it was unclear to me how the main analysis used by the authors - ’topological causality’ - works. Better control situations could probably be used but it is difficult to suggest any without knowing what the analysis does exactly. The authors claim that they are testing causality in some places and mention that they can’t really assess causality in others. However these concerns might disappear with better explanations of the method and its potential limitations.

Summary:

The authors reanalyse an existing dataset containing extracellular freely-moving recordings in the dorsal CA1 hippocampus and basolateral amygdala of rats (Girardeau et al., 2017). The recordings were done during different sleep stages (REM and non-REM) and also while the rats were running between two ends of a linear track; in specific trials, one direction of the run would trigger an air-puff, aversive for the animal. This dataset is well-suited to the study of cross-area communication related to the learning and consolidation of aversive experiences. The authors note that the present study is exploratory and they do not have any a priori hypotheses.

The authors employ a two-step analysis. First, they use a method that seems relatively novel in the neuroscience field, called ’topological causality’ to evaluate the putative influence of one brain region over the other. In this case the method is applied to the power of specific oscillations in the local field potential (delta, theta, gamma, ripple etc.). The authors find specific patterns of interactions between the hippocampus and the amygdala (usually termed ’causal’ in the manuscript) during specific stages of the experiment. The method is compared to previously used ones such as Granger causality and is found to have similar or different outcomes depending on the sleep stage.

In a second part, the authors employ another potentially new method to visualise and classify activity patterns reordered in an unsupervised manner, using a measure called the ’Progress Index’. This method uses as an input the principal components of a data set comprising of 1) the LFP power at the different power bands stated above and 2) the different types of topological causality computed from the LFP. The reordrered data obtained is then formally classified using ’Sapphire-based clustering’ and the performance of the classification method in finding behaviourally-relevant states is evaluated by comparing the clusters to ground-truth labels of the data (when available). The new method is shown to be better than other clustering methods and than chance at finding such clusters for 2 out of 3 rats.

General Strengths & Weaknesses

The authors’ effort to apply these complex methods to a relatively complex data set is impressive and should be applauded. These methods might be of interest to the neuroscientific community which has a growing need for tools allowing unsupervised analysis of large and multi-area datasets. Despite the complexity of these analyses, the process remains relatively understandable, thanks to the didactic figures and explanations provided in the manuscript; however, this could still be improved further. I also appreciated just looking at the Sapphire plots, even though it is difficult to extract any strong conclusion from them. Thus, this manuscript provides a potentially interesting methodological contribution to the field.

On the other hand, the contributions of the manuscript in terms of neuroscientific knowledge (especially beyond that of the original paper, Girardeau et al., 2017) are unclear to me as it does not seem to provide any significantly novel insights about the brain, and it does not test a specific hypothesis (acknowledged by the authors). The authors do propose a new hypothesis at the very end of the discussion (role of the basolateral amygdala beta band in memory consolidation) but, from the wealth of data analysed, one could have expected a lot more. It also has some drawbacks, namely, 1) it is unclear how the ’topological causality’ analysis can provide any information about causality, as no causal manipulation is done in the experiment; 2) most of the results of the unsupervised classification of states of interest can be quite simply explained by the difference of power in theta and ripple band in the different states (high theta in REM/running, low theta in nREM sleep or rest); 3) the results are quite variable across subjects (usually replicating for only 2 of the 3 rats) ; 4) the analysis might not be suited for very fast oscillations such as hippocampal ripples; 5) volume conduction could perhaps explain some of the results. However, points 2 to 4 are already discussed by the authors and the other points might be relatively easily clarified.

In summary, I believe this manuscript is likely to be of interest to the readership of eNeuro looking towards using new analysis methods provided the main concerns are addressed by the authors - detailed below.

Major concerns

1. ’Topological causality’ analysis:

a. While there are quite a few panels attempting to explain how this analysis operates (which do help), the paper would greatly benefit from a more intuitive explanation of this measure in simple terms so that even a naive reader can evaluate its validity. What exactly is it measuring? What does the term ’topological’ stand for in this context?

b. Related to this, it is unclear how this analysis manages to extract ’causality’ between two brain regions from, in this case, the power of LFP oscillations, since there is no manipulation of neural activity. The only explanation I can think of is that the analysis measures how well activity in region X predicts activity in region Y, with some delay supposedly reflecting transfer of neural activity. This is not proving causality as both regions could act this way independently, either by chance, because they are driven by similar sensory stimuli, or because they receive inputs from the same brain region. If this is all that the analysis does, I would suggest that the authors remove the mentions of ’causality’, ’driven by’, ’causal links’, ’causal patterns’ etc. throughout the manuscript, while keeping the term ’topological causality’ as it is the name of the analysis. The authors already discuss this and acknowledge the fact that they cannot be certain about any detected causality, but there are still instances in the manuscript that use the term. Ideally, a control with an obvious absence of causality would be useful, for example using the hippocampal data from one animal and the amygdala data from another, to assess what would be the baseline level of ’causality’ found by the analysis. However, at this stage, it seems simpler to avoid implying findings of causality.

------

a. We added a more intuitive explanation of the method in the Results section (line-353), as well as further explanations in Methods (line-96). The term ’topological’ as well as the term ’causality’ are intimately related to the field of dynamical systems and nonlinear time-series analysis which TC stems from. The meaning of ’topological’ has been clarified in the methods, line-101. For the wider issue of the term ’causality,’ please see below.

b. The terms causal and causality have been removed in most instances. This is simply to avoid confusion related to unclear or field-specific terminology. The underlying difference between the notions of causality in nonlinear time-series analysis and in experimental (intervention-based) neuroscience is now discussed (line-653). Causal and causality were indeed used previously in the sense that is prevalent in the field of nonlinear time-series analyses. This seemed obvious to us as the manuscript deals only with data analysis, but we understand and agree that this might be misleading if one is used to infer causality from direct experimental manipulation. To replace it, we largely resorted to the terms ’directed interactions’ or ’influence’ as adopted also in works dealing with Granger causality.

The most important peculiarity of the ’causality’ in TC and in the dynamical systems field is the following. Briefly, entanglement of the system’s parts becomes possible due to nonlinear, reciprocal coupling. Let us assume that X is affected by Y, which, in turn, is affected by its own history and the coupling with X. Then, the contribution of Y cannot be formally removed from the history of X (and vice versa), and a clear separation between cause and effect is not achievable (non-separability, Sugihara 2012, line-640). What TC tries to measure instead is the time-local asymmetry in apparent reciprocal influence, and it bases its calculation on analytical considerations (Harnack 2017). This becomes a caveat for a causal interpretation if the dynamics are influenced by variables that exert a unilateral influence but are not captured in the observations (we did and do discuss this possibility in the first paragraph of Section 4.1, possibility of “an external signal passing through,” line-663), and mention the entorhinal cortex as one likely source of such signals in Section 4.2 (line-716).

Finally, regarding the additional controls and baseline determination: our TC values are statistically established against such a non-causal baseline already. The shuffling test in conjunction with the Benjamini-Yekutieli correction is a standard and, due to the latter, likely overly stringent way of establishing whether observed TC values exceed the “baseline level” referred to by the reviewer. We thus emphasize that the change in terminology is not due to concerns about the values and their meaning, which is well-defined.

------

2. The possible effects of volume conduction of the different LFP rhythms should be discussed (e.g. Bertone-Cueto et al., 2020, https://www.frontiersin.org/articles/10.3389/fnsys.2019.00078/full “Regions in the convex side, such as visual, auditory and entorhinal cortices, amygdala, colliculus, and even some mesopontine regions would also receive volume-conducted hippocampal theta of smaller amplitude.” ). Could oscillations generated in the hippocampus also be detected in the amygdala? How does the analysis prevent interpreting this as a ’causal’ influence of hippocampus on the amygdala? Fast oscillations like ripples are likely to propagate less, but because the analysis is less appropriate for these oscillations (which are much faster and transient than the timescale used in the analysis), the most interesting findings will be for slower oscillations and these seem more likely to spread through brain tissue.

------

There are some observations that tend to exclude the possibility of volume conduction for theta rhythms from hippocampus and the amygdala. These are summarized briefly in Bocchio 2017 and in Pape and Pare 2010 (pg. 428 for theta and pg. 431 also for gamma, but also Headley 2021). We report here directly the former for simplicity:

"It is still not clear whether LFPs are intrinsically generated within the BLA, are triggered by synaptic interplay from various inputs, or they merely reflect activity that passively propagates from nearby sources; e.g., the hippocampus (Pape and Pare´ , 2010). Yet several lines of evidence suggest that rhythmic activity in the BLA is unlikely to be volume conducted from neighboring areas. Specifically, BLA neurons display intrinsic membrane oscillations at theta frequency range (Pape and Driesang, 1998; Pare’ et al., 1995a). Furthermore, the firing of BLA neurons fluctuates rhythmically with local theta and gamma oscillations (Karalis et al., 2016; Popescu et al., 2009; Stujenske et al., 2014). Finally, theta synchrony between the LA and CA1 hippocampus is observed during fear memory retrieval, but not during exploratory behavior when CA1 displays strong theta activity (Narayanan et al., 2007; Seidenbecher et al., 2003).”

In our data set, we computed the phase difference between the hippocampal and BLA LFPs in order to check whether a zero phase lag was present between the two time-series. The results are shown in Fig. 2-2.

The role of volume conduction for theta, if present, should be negligible since the distributions of phase differences are broad, differ across different epochs, and do not consistently peak at zero. This result does not contrast with Bertone-Cueto 2020 given that the frequencies analyzed there do not go beyond 4 Hz. The expectation for volume conduction are phase-locked signals with a difference that is essentially zero (Nolte 2004). Delta oscillations also show broad distributions but, unlike the theta case, these are showing weak but consistent peaks at zero and do not vary as much across behavioral phases. Taking into account also the results of Bertone-Cueto 2020, we concede that volume conduction can be a factor in the coupling of the delta bands. We thank the reviewer for highlighting this point, which we failed to discuss before. In the hippocampus, unlike the theta band, delta activity is less rhythmic and more irregular and studied less on its own (Schultheiss 2019, Furtunato 2020, Fig.2), with a primary focus on slow-wave sleep and cortical areas (discussed in the introduction of Schultheiss 2019). Little is known also for the delta oscillations in the BLA (Bocchio 2017) and, to our knowledge, studies analyzing specifically the synchronization and/or interactions between hippocampus and BLA are lacking.

Regarding the reviewer’s question about how this would manifest itself in TC, Harnack 2017 showed that ’true’ interactions, in the context of a common underlying drive, are in fact discernible as long as the two time series do not synchronize completely (Fig.4b therein). In our case, this caveat affects primarily the delta-delta TC. Assuming that hippocampal delta oscillations spread by volume conduction, a detected interaction from the delta band of the BLA might indeed be erroneous as they just could just be a ’shadow,’ for lack of a better word, of an internal interaction between hippocampal bands. These considerations and caveats are now acknowledged in both the Results (line-396) and Discussion sections (line-730).

------

3. Is the topological causality measure more likely to find ’increased causality’ in a given band where the power is higher (for example, increased theta during REM sleep compared to nREM sleep)? If yes, how do the authors control for this?

------

The short answer to this is that this is a necessary property of the detection method and not something that can or should be controlled for. With any statistical inference procedure, the confidence will depend on the properties of the data, here primarily the ratio of any variance of interest (coupled changes in different bands and regions) relative to the intrinsic (error) variance. This holds in the same way for the simplest linear measure, time-lagged cross-correlation, as it does for TC. To observe variance of interest of a sufficient ratio, a certain level of power is required, and this coupling between detectability and power is necessary unless the data were to be provided noise-free.

To minimize the impact of systematic differences between signals, Harnack 2017 apply what they call a quantile transformation F(x), which would be more accurately called a rank transformation since it simply consists of replacing data values with their empirical cumulative distribution function (CDF) estimates (also clarified now in the manuscript, line-107). We apply the same to the band power x(t), which offers the advantage that TC values do not depend on the particular shape of the distribution, e.g., cases characterized by long tails for bursty bands like the ripples one. Importantly, this makes the TC calculation invariant with respect to any monotonic transformation of the time series since the empirical CDF will be either, as before, F(x(t)), or it will be 1 - F(x(t)). The latter does not affect the PCA step and the calculation of singular values that quantify the expansion of the mapping from one embedded space to the other (Harnack 2017, SI sec.A).

------

Minor concerns

1. The title and goal of the manuscript could be clarified to ensure that the reader’s expectations are met, perhaps putting more emphasis on the methodological aspect (something like: ’Unsupervised methods for detection of neural states: case study of hippocampal-amygdala interactions’), or, if the emphasis is put on experimental findings, state these.

------

We thank the reviewer for the suggestion. We changed the title as suggested, stressing more the methodological aspect. As mentioned at the beginning of this rebuttal letter, modifications aimed to move the focus to the methodological aspects are found throughout the manuscript.

------

2. While reading the paper it seemed obvious that any classification method using LFP as an input would be able to separate the different states (REM, non-REM, running, eating, etc.) simply from the different patterns of LFP in these different states (especially given that REM and non-REM are labelled using the delta/theta ratio). However, an interesting analysis is shown in Fig6 where the contribution of the TC values is assessed and found to significantly improve the classification (for 2 of the 3 rats). This shows that there is information in the interactions between the two brain regions beyond their independent activity. I feel that this could be emphasized in the introduction instead of having to wait until the last figure to discover it.

------

We agree that this is a result that was not emphasized enough in the text. It is mentioned now in both the Abstract and in the Introduction (line-46). Other observations regarding the contributions of LFPs and TCs are discussed for minor point 17.

------

3. When reading the paper in order (methods first), many concepts are not explained (e.g. what data is actually analysed, what is considered a state, what is a cluster, what is Taken’s theorem, what is time-lagged embedding, what does ’topological causality’ mean, which manifold is referred to L106? etc.). These are mostly clarified once the reader reaches the relevant part in the results, still, an introduction of the different concepts mentioned in the methods when they are first mentioned would be welcome. Several of my other comments below will probably be related to this.

------

Admittedly, we do not fully follow this concern. When reading the methods first, 2.1 and 2.2 did present the experimental setup and how the data are analyzed. Time-lagged embedding was explicitly defined directly in 2.3 where it was introduced. Clusters/states were explicitly defined in the introduction at first mention (disregarding the abstract of course). This is all maintained in the revised version. Naturally, we cannot reproduce the contents of the main reference for TC in full but we have added a qualitative description of Takens’ theorem and TC to Section 2.3., which is the part the reviewer appears to be referring to most in this point. For this revision, inspired by the criticism, we also tried to improve the readability of the results by embedding more of a methodological backdrop in a high-level view.

------

4. Figure 1-1 (and also 4-1): links between what is shown in the figure and what is explained in the legend could be generally improved. If specific terms are used for the first (in the order of the manuscript) time in the legend, they should be explained. When specific signs, lines, colors etc. are used in the figure, they should be explained in the legend.

a. in Fig 1-1, where is m in the figure? What are the differences between time points in the embedding? What is the simplex projection method? Why do the time-series need to be aligned since they are already using a common time reference (t)? What do tau HPC(m-1) and tau BLA(m-1) mean, what do the two thin dotted lines represent?

b. In Figure 4-1: new terms should be explained. “peaks not satisfying specific selection criteria are rejected”: what are these criteria?; “partitions from (3) and (4) are eventually combined”: maybe the authors mean 2 and 3?

------

We thank the reviewer for highlighting these issues.

a. Figure 1-1 is cited for the first time at Fig.1, which is due to the formatting requirements of eNeuro (each supplementary figure has to be cited in the caption of its parent figure). In the text, it is cited for the first time in Section 2.3 as it is tightly related to the alignment analysis. This mention appears only after all the necessary information has been provided. We modified the legends of Figs. 1 and 1-1 to cite the sections of the text necessary for the understanding of the concepts and symbols present. In addition, minor modifications have been made in the caption of Fig.1-1 to improve clarity.

b. The image is taken from the original SbC publication. All details of the method are provided there, which is now explicitly cited. We added small clarifications to the legend to facilitate the understanding.

”...maybe the authors mean 2 and 3?” -> Corrected, thanks!

------

5. In figure 4-1’s legend, why is the significance threshold chosen to be p<0.01 while it is p<0.05 in all other analyses?

------

This is the threshold set in the original SbC clustering method that has been published here. In our case, a higher value (p<0.05) leads to the addition of only one or two barriers and, thus, clusters, which does not affect our results.

------

6. L75 “Only LFPs from the right basolateral amygdala (BLA) were used in the analysis”: why is this the case and how might this influence the analysis? Are the hippocampal signals bilateral? Does this change between rats?

------

The available hippocampal signals are unilateral, either left or right depending on the rat (as written in the original paper, the precise localization is not found in the data set). In a preliminary analysis conducted on Rat2, we found no relevant differences in the TC values computed. These results are shown in Fig. R1.

See Fig.R1 in https://gitlab.com/CaflischLab/unsupervised_hpc-bla/-/blob/master/docs/rebuttal_figures.pdf

Differential contributions between right and left amygdala have been observed in neuroimaging studies in humans (Dyck 2011, Baeken 2014) and in rats (Blair 2005, Baker & Kim 2004). In the first rodent study (Blair 2005), it is suggested based on a Pavlovian fear conditioning experiment that CS-US (conditioned and unconditioned stimuli) association memories rely on the amygdalar region contralateral to the aversive stimulus (only one eyelid is subjected to a shock). Our aversive stimuli are somewhat lateralized, that is, the air puffs are manually delivered approximately perpendicular to the linear track, but their incoming side with respect to the rat depends on the specific session that is analyzed (the air puff occurs only along one direction of motion). In the second study (Baker & Kim 2004), inactivation of the right amygdala implicates stronger memory deficits in retention of fear conditioning memories of context. However, differences between the inactivation of the two sides were not observed in pretraining lesions nor in conditioned fear to an auditory stimulus.

These results hint at differences in functionality between left and right sides, yet we could not find follow-ups on this topic from recent years. Even though literature results suggest the potential for a differential role performed by the two sides of the BLA, we decided to stick with one side only for all the rats (right), and we did not try to either combine them or to alternate this choice across rats. The reasons are the following: (1) a translation to the experimental modalities we analyzed is not straightforward and impractical to implement due to the session specificity; (2) we could not establish an apparent difference in preliminary results (Fig. R1); (3) the right side offered two recording sessions more than the left one in total.

------

7. L79 could the authors make it extra-clear which of the manuscripts’ rat names correspond to the names used in the original paper?

------

We added an explicit association between our names, the names in the original paper, and those that effectively appear in the data set (line-66).

------

8. L86: ’the identification of different sleep phases (NREM/REM) was done with the help of a k-means clustering of the ratios of the delta to theta powers in the hippocampus’ Could the authors indicate which ratios (higher theta or higher delta) correspond to which sleep phase (NREM or REM)?

------

Higher ratios between delta and theta point to NREM states, and, vice versa, lower ratios point to REM. We clarified this in the text (line-74).

If the reviewer is interested in the presence of theta power during NREM sleep, we discussed this at length with one of the original reviewers (see reuploaded rebuttal letter, last point of R2; also Girardeau 2017, Fig.2b).

See discussion of the previous rebuttal letter in https://gitlab.com/CaflischLab/unsupervised_hpc-bla/-/blob/master/docs/rebuttal_figures.pdf

------

9. L87 why are the frequency limits for the same rhythm different for different brain regions (e.g. theta in hippocampus is 7-14Hz but 4-12Hz in BLA)? It looks like an explanation for this is given L311 (“in order to bridge distant frequency bands [...] we homogeneize the intrinsically heterogeneous timescales of variations in the lfps across the different band”), however, this explanation could be clarified.

------

For the BLA, we chose a typical frequency range reported in the literature while for the hippocampal theta band we followed Girardeau 2011. In particular, the latter choice was made with regards to neuronal activity embedded within the theta cycles as we planned to investigate also spiking data. This range was kept throughout the analysis even though, admittedly, it would have been more consistent to keep it equal with the BLA one. As a simple sanity check, we extracted the theta power in the 4-12 Hz range during 1h of preSleep phase in 6 sessions (2 per rat). The Pearson correlations between this signal (4-12 Hz) and the one used in the manuscript (7-14 Hz) all ended up higher than 0.88. The relevant literature references motivating our choices have been added to the text.

The quoted sentences “We have chosen the band powers in order to bridge distant frequency bands...” refers primarily to the utilization of band powers per se to combine distant frequency bands, like delta and gamma, in a single data set. It does not deal with the difference in theta (and, marginally, beta) frequencies between BLA and hippocampus.

------

10. L241 refers to two axes, but there is no figure referred to. Maybe the authors could refer to the example figure (4-1)?

------

Thank you for noticing this. We added the reference to Figure 4-1b, panel 2.

------

11. L288 what does Mds mean?

------

Mds refers to “multidimensional scaling”. This acronym was, by accident, defined only later in the Results section. It is now fixed (Methods Sec. 2.12), thank you.

------

12. L 322-323 “TC values that do not pass this significance threshold are zeroed explicitly (P-values <0.05)” perhaps the authors mean p-value>0.05?

------

Corrected. Thanks.

------

13. In the example in Figure 1: “The actual values (solid lines) exceed the chance level [...] only for short, sporadic intervals” -> it would help to add stars or arrows to highlight these intervals.

------

Bars have been added on top of the plots to facilitate the identification of these significant intervals.

------

14. L331 “low frequency bands”: please detail at least once, the first time they are mentioned, which of the bands are considered low-frequency. Also, it should probably be hyphenated (low-frequency bands’).

------

The term includes delta and theta, and, depending on the context, also beta. In any case, whenever this term is used, the exact bands are specified immediately after.

------

15. L377-378 “we partitioned the NREM data into patterns featuring SWRs and those that do not” It is mentioned later that the SWR timestamps were provided in the original data set, but please explain how they were detected.

------

A brief description of the identification procedure has been added to Section 2.1 (line-79).

------

16. It would be informative to add a line for LFP in the theta band, or theta power, in figure 4 and if possible the other Sapphire plots (theta is likely to be a very strong predictor of the different states found).

------

We added an annotation showing the z-scored theta power of both hippocampus and BLA to all of the Sapphire plots.

------

17. L 434 “Comparing across animals, we can identify a similar set of basins for Rat2”: I do not find this very convincing - the plots seem quite different across animals. The only constant is that theta and ripple power influence the clustering. Maybe the authors could explain in more detail the similarities across rats and in what way these similarities go beyond differences in LFP characteristics.

------

We added colored bars on the bottom of the Sapphire plot of Rat3 to identify a set of four coarse states (described in the text) that are potentially shared with other rats. SbC clusters are now associated to each coarse state, following a procedure described in the new Section 2.12. Similarities between the rats are detailed near line-501.

As for the power in the theta and ripple bands, they clearly influence the clustering but their presence cannot fully explain the coarse states. The contribution from TCs is apparent by comparing the Sapphire plots of Fig. 4 and Fig. R2 (both Rat3). The latter has been obtained from the data set composed only of LFPs (the corresponding stress value is actually the one shown in Fig.6a). In this plot, it is harder to delineate states from the kinetic annotation, and the TCs are clearly relatively homogeneous across the entire progress index. On the contrary, the four macrostates in Fig. 4 show at least three different values/patterns of average TCs. The difference between Figs. 4 and R2 emphasizes that TCs contribute toward a meaningful clustering. The fact that the plot is annotated with particular features of interest should not lead to the misconception that these are the only features that determine the plot.

See Fig.R2 in https://gitlab.com/CaflischLab/unsupervised_hpc-bla/-/blob/master/docs/rebuttal_figures.pdf

The contribution of TCs is similarly apparent for the other rats. For example, it is primarily TC rather than band power that distinguishes the red from the yellow state(s) in Figs. 4-2 and 4-3.

The leftmost basin in Fig. R2 is a good illustration for this result: that LFPs alone can mask the complexity of the animals’ states. Here, basically all of the NREM points are contained, suggesting that NREM sleep is a neurally homogeneous state. This contrasts with the evidence for neural reactivations, which the yellow state in Fig. 4 clearly alludes to: the annotations indicate subtle differences in SWRs and, less so, theta power to the red state, which cluster NREM points with data points from the Run/Wake epochs.

------

18. Figures with Sapphire plots for the different rats: it would help cross-rats comparisons if the axes shared a similar scale across rats (e.g. speed axis goes up to 60 for rat 3 but 30 for rat 2).

------

The axes related to the panels “SWRs", “Speed", “Hpc rate", “BLA rate” and “TC” now share the same scales across the six Sapphire plots (Figs. 4 and 4-2 to 4-7). The z-scored “Theta power” band has been thresholded to 5 (it peaks up to 10 in some cases).

------

19. How is the starting point of the ’Sapphire plot’ determined? Isn’t the choice of start point likely to change a lot the states / basins found?

------

The starting point of the progress index (PI) is chosen as the central point of the largest cluster (in terms of the number of points it contains) extracted from the hierarchical clustering that underlies the creation of the short spanning tree (and resultant PI). We added this clarification in line-221. This choice stems from the experimental observation that the PI tends to rapidly reach/descend into the largest state(s) regardless of the initial point chosen. This is due to the fact that the largest state(s) is also usually one of the densest (lower distances between points), and the procedure used to construct the PI follows a minimum distance criterion. Based on our extensive use of the method, on this and on other data sets, we observed that the choice of the initial snapshot affects just the ordering of the states/basins along the PI (which is not meaningful) but not their existence and identification per se. The rearrangement of the SbC clusters within the Sapphire plot described in line-255 was designed to reduce this variability in the ordering.

------

20. L451 How does SbC find clusters and number of clusters?

------

The SbC procedure is summarized in Section 2.8 and described in Fig. 4-1. The full details of the method are provided here, with various applications and a comparison to other methods. The number of clusters cannot be set directly but it does depend monotonically on the bin size of the histogram along the PI axis (Section 2.8, wPI) (the lower the size the higher the number of clusters found). As explained in Section 2.8, the value was selected in order to find a compromise between a manageable number of states (<∼ 100) and a sufficient density within each bin. We added a few sentences at line-526.

------

21. Figure 5

a. legend: it is a bit unclear how the affinity scores are computed from that figure.

b. In plots b-d, how are the labels associated to some of the clusters? Why do only some clusters have labels?

------

a. We added in the caption a clarification on how the scores were computed.

b. The peculiarity of the unfolding technique is that both labels/judges (11) and clusters (∼90, for each rat) are projected at the same time, thus yielding a total of ∼101 points in each of the Figures 5b-d. This is described in Fig. 5a (panel 4) since labels and clusters appear as rows of the same reduced data set (Mds coordinates).

In a typical dimensionality reduction setting, the procedure consists in: (1) using the affinity scores as the feature data set, i.e., each label (column) corresponds to a dimension, (2) computing a distance matrix between the clusters (rows), (3) plugging the distance matrix into the dimensionality reduction algorithm (in this case a multidimensional-scaling). In the unfolding technique, the affinity matrix itself constitutes a block of the distance matrix, namely, the block related to distances between clusters and judges/labels, while the other blocks remain empty (unused). This implies, in practice, that the affinity matrix is directly plugged into the stress computation (Eq. 3).

We added a short and a longer explanation in the caption and in the Results section (line-542), respectively, on how the unfolding is performed.

We labelled only the points related to the labels/judges (red points); the remaining ∼90 points were colored based on their PI position (the alternative with the cluster numbers would clutter the plot and be largely uninformative to the reader).

------

22. L472 “it is clear that the placement does show an affinity with specific labels, which is highlighted by the coloring according to PI position as seen”: this is not very convincing (different colors seem to be all over the place), perhaps the authors could describe better what they mean.

------

A systematic distribution of colors is clearly visible for Rat2 and Rat3 while for Rat1,which might have been what the reviewer focused on, the distribution is indeed more disordered. We added clarifications in this regard in the vicinity of line-555.

------

23. The authors could discuss what makes rat 2 different from the others in terms of contribution of TCs to the clustering. Were the recording probes placed in different locations? Was the behaviour different?

------

As discussed in minor point 6, we do not have information on the recording site of the hippocampal probe (dorsal CA1) whereas the amygdala probe analyzed is always the BLA right (lowered every day, as described in Section 2.1). We could not find any outstanding differences between Rat2 and the other rats in terms of behavior (number of air puffs, rewards) or in sleep (distribution of wake, NREM or REM), in distributions of the power bands or in the average TC values for the 72 combinations.

We note that with an alternative preprocessing of the data (description in Section 2.6, Sapphire plots in Figs. 4-5, 4-6, 4-7) the contribution of TCs to the clustering becomes significant for Rat2, while worsening for the others. Given this, we have to assume that different preprocessing pipelines affect the rats differently, stressing in one case TC more than in the other, and, consequently, different preprocessing pipelines should be tried for each animal. These considerations are now reported at line-606.

------

24. L 590: ’tcs do go beyond linear models of causation like GC and CCF”: how?

------

We refer to the fact that linear techniques like GC and CCF cannot address the non-separability issue present in nonlinear systems. This point is related to major point 1. The mentioned sentence has been made clearer in the text (line-665).

------

25. L656 “they seem to provide a gating mechanism”: I’m not sure where this hypothesis comes from (it is not mentioned in the cited references). I suggest removing ’gating’.

------

We removed “gating.”

------

26. Paragraph L669: It is unclear what is the point of this paragraph. The authors describe in qualitative terms the 4 states visually observed in one of the figures (for one rat) but do not attempt to explain what could be the functional relevance of these states; instead, they provide a relatively vague and rather obvious conclusion: “these causality patterns hint at memory consolidation mechanisms involving neuronal subpopulations.” Would this conclusion have changed had the authors found a different number of states or different types of states? What makes these states unique and interesting?

------

We replaced this final sentence with a few considerations regarding the original work and the potential use of spiking data (line-795).

------

27. While the manuscript is generally very well written, there are a few typos eg. Legend of Figure 1-1 “The differences between time points in the embedding*are* dependent” ; L588 “have to interpreted” -> have to be interpreted.

------

Corrected. Thank you for noticing these.
